# Evaluating DREAMS HIV prevention interventions targeting adolescent girls and young women in high HIV prevalence districts in South Africa: protocol for a cross-sectional study

**DOI:** 10.1186/s12905-019-0875-2

**Published:** 2020-01-16

**Authors:** Gavin George, Cherie Cawood, Adrian Puren, David Khanyile, Annette Gerritsen, Kaymarlin Govender, Sean Beckett, Mary Glenshaw, Karidia Diallo, Kassahun Ayalew, Andrew Gibbs, Tarylee Reddy, Lorna Madurai, Tendesayi Kufa-Chakezha, Ayesha B. M. Kharsany

**Affiliations:** 1 Health Economics and HIV and AIDS Research Division (HEARD), 4th Floor, J Block, Westville Campus, University of KwaZulu-Natal, Durban, South Africa; 2 Epicentre AIDs Risk Management (Pty) Limited, Paarl, South Africa; 3 Centre for HIV and STIs, National Institute for Communicable Diseases, National Health Laboratory Service (NICD/NHLS), Johannesburg, South Africa; 4 U.S. Centers for Disease Control and Prevention (CDC), Pretoria, South Africa; 5 Gender and Health Research Unit, South African Medical Research Council (SAMRC), Pretoria, South Africa; 6 Biostatistics Research Unit, South African Medical Research Council (SAMRC), Durban, South Africa; 7 Global Clinical and Virology Laboratory, Amanzimtoti, Durban, South Africa; 8 Centre for the AIDS Programme of Research in South Africa (CAPRISA), University of KwaZulu-Natal, Durban, KwaZulu-Natal South Africa

**Keywords:** HIV incidence, HIV prevention, Adolescent health, South Africa, DREAMS, AGYW

## Abstract

**Background:**

Young women in sub-Saharan Africa remain at the epicentre of the HIV epidemic, with surveillance data indicating persistent high levels of HIV incidence. In South Africa, adolescent girls and young women (AGYW) account for a quarter of all new HIV infections. Determined, Resilient, Empowered, AIDS-free, Mentored and Safe (DREAMS) is a strategy introduced by the United States President’s Emergency Plan for AIDS Relief (PEPFAR) aimed at reducing HIV incidence among AGYW in 10 countries in sub-Saharan Africa by 25% in the programme’s first year, and by 40% in the second year. This study will assess the change in HIV incidence and reduction in risk associated behaviours that can be attributed to the DREAMS initiative in South Africa, using a population-based cross-sectional survey.

**Methods:**

Data will be collected from a household-based representative sample of AGYW (between the ages 12–24 years) in four high prevalence districts (more than 10% of the population have HIV in these districts) in South Africa in which DREAMS has been implemented. A stratified cluster-based sampling approach will be used to select eligible participants for a cross-sectional survey with 18,500, to be conducted over 2017/2018. A questionnaire will be administered containing questions on sexual risk behaviour, selected academic and developmental milestones, prevalence of gender based violence, whilst examining exposure to DREAMS programmes. Biological samples, including two micro-containers of blood and self-collected vulvovaginal swab samples, are collected in each survey to test for HIV infection, HIV incidence, sexually transmitted infections (STIs) and pregnancy. This study will measure trends in population level HIV incidence using the Limiting antigen (LAg) Avidity Enzyme Immuno-Assay (EIA) and monitor changes in HIV incidence.

**Discussion:**

Ending the HIV/AIDS pandemic by 2030 requires the continual monitoring and evaluation of prevention programmes, with the aim of optimising efforts and ensuring the achievement of epidemic control. This study will determine the impact DREAMS interventions have had on HIV incidence among AGYW in a ‘real world, non-trial setting’.

## Background

Adolescent girls and young women (AGYW) in sub-Saharan Africa remain the sub-population most susceptible to HIV infection [1]. In 2017, approximately 65% of all new HIV infections occurred in sub-Saharan Africa [2], with AGYW accounting for 74% of these infections, with more than 1000 HIV acquisitions amongst adolescents occurring daily [2, 3]. South Africa (SA) currently has the largest HIV epidemic in the world, with an estimated 7,520,000 people living with HIV (PLHIV) in 2018 [4]. A National surveillance study in 2017 estimated that 20.0% (95% CI 18.7–21.4) of South Africans fifteen years and older, were living with HIV [5] . In 2017, the prevalence amongst males was lower compared to females (4.7% versus 5.8% among those aged 15–19 years, and 4.8% versus 15.6% among those aged 20–24 years). The highest HIV-incidence (1.5%) is amongst South African females aged 15–24 years compared to 0.5% amongst young men their own age, translating into over 66,000 new infections amongst AGYW in South Africa (SA) in 2017 [5].

Several factors contribute to the observed increased rates of HIV acquisition amongst AGYW. Young women are biologically more susceptible to acquiring HIV than men. The risk of HIV transmission during unprotected vaginal intercourse is greater for women than men; and the risk for AGYW is further heightened because their vaginal tracts are immature and tissue tears occur more easily from sexual activity [6]. Furthermore, a study concluded that women with genital inflammation had an almost three-fold higher risk of HIV acquisition [7]. The risk of acquiring HIV also increases in the presence of sexually transmitted infections (STIs), with the majority of STIs occurring in young people under 25 years of age [8–10]. The estimated prevalence of STIs in South Africa among female clinic or community populations aged 15 to 24 years old was 20.6% for chlamydia, 8.9% for gonorrhoea, 20.0% for trichomoniasis, 53.7% for HSV-2, and 52.4% for bacterial vaginosis [11].

Compounding the prevalent biological factors, structural factors further facilitate HIV transmission amongst AGYW [12–14]. Many AGYW, enter into sexual relationships with older partners as a result of socioeconomic deprivation [15], with age-disparate relationships found to increase HIV-acquisition amongst AGYW [16–19]. Age disparate relationships increase AGYW’s susceptibility to HIV acquisition, because older men are more likely to be HIV positive [8, 20, 21], whilst these partnerships are also characterised by inconsistent condom use [22–24] and concurrent sexual partnering [25]. Relationships are also more likely to be transactional in nature [26], which has been demonstrated to also increase HIV acquisition [27]. In addition, AGYW are vulnerable to intimate partner violence (IPV) and dominant male partner behaviours, both of which increase their susceptibility to acquiring HIV [28].

In response to the high and sustained HIV-incidence among AGYW, interventions have been developed and implemented targeting this particular population cohort. The Determined, Resilient, Empowered, AIDS-free, Mentored and Safe (DREAMS) initiative is a comprehensive, multi-sectoral strategy introduced by the United States of America (U.S.) President’s Emergency Plan for AIDS Relief (PEPFAR) in 2014. DREAMS initiatives exist in 10 countries in sub-Saharan Africa: Kenya, Lesotho, Malawi, Mozambique, South Africa, Swaziland, Tanzania, Uganda, Zambia, and Zimbabwe. The initiative consists of evidence-based interventions, aimed at addressing the structural and behavioural drivers that increase AGYW risk of acquiring HIV, including poverty, gender inequality, gender-based violence (GBV), absence of parental and community support, and lack of education and vocational training [29]. The target of the DREAMS initiative is to reduce HIV incidence by 25% amongst AGYW in targeted high-prevalence areas by the end of the first year of implementation and by 40% at the end of the second year of implementation [30].

Each DREAMS country has identified context relevant interventions to include as part of an evidence-based approach to HIV prevention. In South Africa, DREAMS interventions are rolling out in five high HIV burden districts, namely: City of Johannesburg, Ekurhuleni, eThekwini, uMgungundlovu and uMkhanyakude. The DREAMS initiative is executed through existing U.S. government funded programme implementers including community, faith-based and non-governmental organizations, referred to as implementing partners, along with South African Government structures operating at the provincial, district, sub-district, metro, ward and village level. DREAMS aims to scale-up impactful and evidence-based interventions targeting AGYW, in an effort to bring the HIV/AIDS epidemic under control.

The DREAMS suite of interventions includes condom promotion and provision, HIV Counselling and Testing (HTS) and linkages to care, pre-exposure prophylaxis (PrEP) (targeting sex workers specifically), GBV prevention as well as post-violence care and support; linkage to sexual reproductive health (SRH) services, comprehensive sexuality education and social asset building. DREAMS initiatives aim to strengthen school-based HIV and GBV prevention efforts, together with strengthening community mobilisation and norm change efforts through parenting and caregiver programmes, social protection initiatives and interventions specifically targeting male sex partners. The age range of participating AGYW varies across these different activities. For example, 10–14 year olds are not included in condom promotion activities, while PrEP is provided to young women aged 20–24 years who engage in sex work [31]. Whilst younger girls together with boys and men are targeted by some of the aforementioned interventions, this study focuses on females aged between 12 and 24 years of age. The evaluation will provide data on the impact of the DREAMS interventions and will inform future development and potential expansion of these interventions targeting adolescent girls and young women.

### Primary hypothesis

The DREAMS initiative will collectively reduce HIV incidence amongst AGYW by 25% by the end of year one of its implementation, and by 40% by the end of year two in those districts where DREAMS programmes were implemented.

### Study aim

The aim of this study is to determine the impact of the DREAMS initiative on HIV incidence amongst a household-based representative sample of AGYW (12–24 years) in four of the five districts in KwaZulu-Natal (KZN) and Gauteng (GP), South Africa. An HIV incidence baseline will be estimated primarily using incidence findings from existing studies. HIV incidence in the South African National HIV Prevalence, Incidence, Behaviour and Communication Survey Study (SABSSM) used a similar incidence bio-marker-based methodology to one adopted for this study. The HIV incidence findings for AGYW 15–24 years at the national level was 2.54% (95% CI 2.04–3.04) in 2012. An earlier longitudinal study on HIV uninfected 14–30 year old women, undertaken between March 2004 and May 2007, revealed that HIV incidence in young girls below the age of 18 years was 4.7 [95% Confidence interval (CI) 1.5–10.9) compared to 6.9 (95% CI 4.8–9.6)/100 women years (wy) in those aged 18 - 30 [32]. The HIV Incidence Provincial Surveillance System (HIPSS) situated in uMgungundlovu, KZN, included two cross-sectional surveys between June 2014 and June 2015 and between July 2015 and July 2016. The HIPSS study population was drawn from uMgungundlovu, the same area for this study. The HIPSS incidence in adolescent girls 15–19 years was 4.63% (95%CI, 3.29–6.52) and 4.00% (95% CI, 2.74–5.85) for women 20–24 years old in the 2014 survey [33].

### Study objectives

The primary objective of this study is:

- Measure the change in HIV-1 incidence following implementation of the DREAMS suite of interventions within a ‘real world, non-trial setting’.

The secondary objectives are to:

- Measure HIV prevalence, and proportion of HIV positive AGYW on ART and ART naïve with detectable and undetectable HIV-1 Ribonucleic Acid (RNA) viral load.
- Measure prevalence of pregnancies and sexually transmitted infections (STIs) including Hepatitis B virus (HBV), Herpes simplex virus type 2 (HSV-2), syphilis, human papillomavirus (HPV*), Chlamydia trachomatis*, *Neisseriae gonorrhoea*, *Trichomonas vaginalis* and *Mycloplasma genitalium.*
- Measure risky sexual behaviour, specifically, the number of past year sexual partners, condom use, and age-disparate and concurrent partnering.
- Measure the prevalence of intimate partner violence.
- Measure performance on selected academic milestones (e.g. academic progress, school completion rates).
- Measure the economic empowerment of AGYW, socio-economic status of their family and selected parent/guardian/caregiver indicators.
- To measure the exposure to the DREAMS programme including condom promotion and provision (male and female); HIV testing services (HTS) and linkage to care; GBV prevention and post violence care and support; comprehensive sexual reproductive health rights (SRHR) services; social asset building interventions; school-based HIV and violence prevention; community mobilization and norms change; parenting/caregiver programmes.

## Methods/design

### Study setting

The four study districts (City of Johannesburg, Ekurhuleni, uMgungundlovu and eThekwini) consist of an estimated 12,073,421 individuals. uMkhanyakude, the fifth DREAMS district, is not part of this evaluation.

The province of KZN remains the worst affected by HIV with an overall prevalence of 15% amongst 15 to 49 year old inhabitants in 2015 [34]. The two districts of interest within KZN, uMgungundlovu (20% HIV prevalence in 2016) and eThekwini (16.8% HIV prevalence in 2016) are among those with the highest HIV prevalence in South Africa [34]. eThekwini contains the third-largest city in the country (Durban), which is the busiest port on the African continent and the main economic hub within the province of KZN [35]. About 68% of eThekwini is considered rural and 32% urban.

uMgungundlovu is the second largest district in KZN after eThekwini and is located in central KZN. uMgungundlovu includes traditional settlements or farmlands, informal, rural and urban settlements. Its local municipalities are largely rural, while the Msunduzi Municipality is highly industrialized. The largest city within the uMgungundlovu district is Pietermaritzburg, which is also the capital of KZN.

The Gauteng province (GP), whilst geographically the smallest, is the most populous province in South Africa. GP has the fifth highest provincial HIV prevalence in the country with a prevalence of 11.1% in 2016 [34]. The HIV prevalence in the two study districts, City of Johannesburg and Ekurhuleni is 11.1 and 14.3% respectively [8]. These districts are densely populated. Whilst highly urbanised with a diverse economy, large urban townships including Katlehong and Tokoza which form part of the Ekurhuleni district. The HIV prevalence amongst AGYW in the City of Johannesburg and Ekurhuleni was estimated to be is 3% in 2012 [36].

### Study design

This study is a single cross-sectional survey targeting 18,500 AGYW, to be conducted in 2017/2018 in four selected districts in South Africa. AGYW between 12 and 24 years will be selected using a stratified cluster-based sampling approach, with districts representing strata and the cluster or primary sampling unit being the small area layers (SALs). SALs were created by merging adjacent Enumeration Areas (EA), which had less than 500 people, to create a spatial area layer that corresponded as closely as possible to the EA layer. The following criteria were used for the establishment of the SALs. EAs were merged if they fell within the same sub-place (a higher order geographic level); EAs were merged if they had the same geographical properties; an EA was merged if its population that resided there was less than 500 people; and lastly, the resulting SALs must have a population total of 500 or more [37].

Stratified sampling was chosen as it reduces sampling error and ensures a greater level of representation when compared to simple random sampling [38]. It also ensures more precise estimates, provided that there is homogeneity within strata and heterogeneity between strata [38]. A questionnaire will be administered and biological samples collected (two micro-containers of blood for dried blood spot (DBS), plasma specimens and self-collected vulvo-vaginal swab samples (VVSS) to test for HIV infection, HIV incidence, STIs as well as pregnancy respectively).

Additionally**,** we will conduct 64 focus groups discussions (FGDs). Thirty two in one district in GP (city of Johannesburg) and thirty two in one district in KZN (uMgungundlovu). The districts were randomly selected. Individuals exposed to DREAMS interventions will be recruited to participate in the study. The FGDs in each district will include: 1) adolescent girls 16 to 18 years old (*n* = 8), 2) adolescent girls and young women 19 to 24 years old (*n* = 8) 3) young men aged from 25 to 35 years old (*n* = 8), 4) stakeholders who support adolescent girls and young women such as teachers, nurses and police men (*n* = 4) and 5) DREAMS implementation partners (*n* = 4). This qualitative component will provide additional insight into the impact of DREAMS interventions on AGYW in relation to the study objectives whilst also identifying facilitators and barriers to accessing the DREAMS interventions. The study followed the Strengthening the Reporting of Observational Studies in Epidemiology (STROBE) guidelines (See Additional file 1).

### Study population

According to 2011 census data [39] the four study districts consist of an estimated 3,679,700 households containing approximately 1,633,906 AGYW. Additional characteristics of the population within the selected districts are listed in Table 1.

**Table 1:** Characteristics of the population within SA DREAMS IE districts (2011–2017)

	eThekwini	uMgungundlovu	City of Johannesburg	Ekurhuleni
Population Size: 10–24 years AGYW [39]	500,214	153,722	584,104	395,866
Population density per square km [39]	1501.90	113.90	2695.90	1652.00
HIV ANC Prevalence (%) [40]	41.1	42.5	27.5	33.5
People living with HIV (PLHIV) [8]	516,167	225,284	533,960	468,521
Number of AGYW 10–19 years receiving grants^a^ [41]	64,716	84,942	189,845	155,135
Number of Orphans [41]	86,734	42,000	61,634	55,261
Number of Girls in schools Ages 10–20 years [42]	238,544	74,602	276,185	161,182
Teenage pregnancy (deliveries at facility in women under 18 yrs. and not terminations) (%) [31]	7.0	8.5	3.6	6.2
Number of SALs	300	150	300	300
Number of households	956,713	272,666	1,434,856	1,015,465

Notes: ^a^In this context, a grant refers to the social assistance provided by the South African Social Security Agency in the form of different grants for children (for example Foster Child Grant, Care Dependency Grant, Child Support grant and Grant-in-aid)

### Sampling strategy

A stratified cluster-based sampling design has been adopted, specifically:

- The four districts will be considered as the primary strata.
- In order to reach 18,500 AGYW in each survey, a total of 1050 SALs will be selected and distributed between KZN and GP, proportional to the targeted sample size in each province (i.e. 10,500 AGYW in GP and 8000 in KZN), resulting in 450 SALs for KZN and 600 for Gauteng. A total of 300 SALs will be selected from eThekwini and 150 from uMgungundlovu; 300 from the City of Johannesburg and 300 from Ekurhuleni, this is proportional to the population size of the districts. uMgungundlovu is less than one third the size of eThekwini, however to ensure that estimates are reported with a higher level of precision for uMgungundlovu SALs were allocated in the ratio of 2:1 between uMgungunglovu and eThekwini.
- To achieve a minimum of 18 AGYW per SAL, taking into account that approximately 1 in 2 households have an eligible individual, and a non-response rate of 20%, 55 households will be randomly chosen within each SAL in order to attain a household-representative sample of AGYW [43].
- All eligible AGYW in the household will be approached for inclusion in the study.

The SAL sampling frame has been triangulated using 2011 census data [39], the 2007 Community survey data [38] and aerial imaging to determine the number of households and population estimate at the SAL level. The SAL sampling frame will be checked across these three data sources and estimates calculated according to province and district boundaries, whilst the population will be further classified according to race, gender and age. To adjust for unequal probability of selected SALs, non-response and to facilitate the interpretation of results at the provincial level, sampling weights will be used in all analyses. From a total of 19,366 SALs across the study area, approximately 1050 SALs will be selected to reach the targeted sample size. The sample size is based on the required number of AGYW (i.e 18,500) and working upwards to determine how many households need to be approached to realise this sample size. If one in two households have an AGYW [39], to obtain 18,500 we would need to approach 37,000 households (assuming they all agree to participate). However, we anticipate that 20% of households will not agree to participate and about 12.5% are unoccupied or invalid (so anticipate approaching 55,000 households).

Study staff will identify households using a Global Positioning Systems receiver to record the geographic coordinates of each randomly selected household. Fieldworkers will visit approximately 55 households from each SAL. Recruitment will continue until the required number of study participants are enrolled. Should a selected household be abandoned, refuse to complete the composition form or should the members be away for an extended period, the next household on the sampling frame will be approached.

The required number of SALs for the evaluation will be randomly selected. Sufficient oversampling of households will be done to avoid repeated sampling of households.

### Eligibility criteria

All females aged 12–24 years residing in sampled households and who are willing to participate in this cross-sectional study and undergo the study procedures including an HIV test, will be included in the study. People who do not speak English, Zulu, Sotho, Afrikaans, who have cognitive or mental challenges (based on the assessment of the participant’s ability to comprehend the study information provided) and those who are deaf and/or mute will be excluded from the study. Individuals in institutions (hostels, prisons and hospitals) will not be eligible for participation in the study.

### Statistical considerations and sample size calculation

The sample size of 8000 per survey in the two KZN districts will have 80% power to detect a reduction of 40% in the HIV incidence rate at the 5% significance level given a false recent rate (FRR) of 0.01%, HIV prevalence of 25%, and an initial HIV incidence rate of 4 per 100 person years (py) [33]. A sample size of 10,500 per survey in the two GP districts will have 80% power to detect a 40% reduction in HIV incidence given a FRR of 0.01%, HIV prevalence of 25% and baseline incidence of 3/100py amongst AGYW aged between 12 and 24 years [8].

The aforementioned sample size calculations are based on detecting change in incidence and not the precision of individual estimates. However, the sample size of 8000 in KZN is adequate to estimate an HIV incidence rate of 4% with a coefficient of variation of 12%, corresponding to a confidence interval of (3.1–4.9%). The sample size in Gauteng of 10,500 will allow us to estimate the HIV incidence rate of 3% with a coefficient of variation of 12% and confidence interval of (2.28–3.72).

### Study procedures

#### Recruitment

The study team will establish Community Research Support Groups (CRSG) in all the study districts following the model adopted by the HIV Incidence Provincial Surveillance System (HIPPS) in the uMgungundlovu district [44]. Data collection will be preceded by building strong community relationships through extensive community engagement and advocacy with local stakeholders including traditional leaders, DREAMS implementing partners, other public, private and NGO service providers within the study districts related to education, psycho-social support, socio-economic support as well as health services. The CRSG will provide a forum for the engagement between researchers and community members, preserving the community’s best interests and ensuring that the study team are aware of any concerns about the research. As with HIPSS, the CRSG will provide input into the informed consent procedures, questionnaire design and administration of the surveys [44].

Following community sensitisation, study staff will approach households included in the sample and introduce the study to the household residents. All individuals in the household who meet the eligibility criteria and are available to be interviewed will be asked to participate in the study. Those who do not wish to participate in the study will be asked to provide feedback about their decision for declining in order to characterise the impact of refusal on the study’s outcomes. Those who agree to participate will be asked to designate a location, either inside or outside their residence, where the survey instruments can be administered and biological samples collected privately.

Participants in the study will be compensated nominally for their time with a gift to the value of between 2 and 3 United States Dollars. A Parent/guardian of AGYW aged 12–17 years will be asked to complete a caregiver questionnaire (See addional file 2). No biological samples will be collected from caregivers, defined as a parent or guardian who is primarily responsible for the pre-selected AGYW’s welfare, specifically the person who prepares meals, seeks medical attention for the AGYW in the case of illness and otherwise cares for the AGYW.

In the situation where the respondent is 17 years or younger and does not live with any caregiver (a child-headed household), a caregiver survey will not be administered. In instances where no caregiver survey is administered, additional questions from the caregiver questionnaire are included in the AGYW questionnaire. In the case of a person being a caregiver to more than one study participant, this caregiver will be required to complete the relevant questions for all AGYW residing within the household. If the caregiver is not present at the time the interviewer accessed the household, the interviewer will obtain the contact details of the caregiver, if available, and schedule a return visit at an agreed upon date and time to obtain informed consent and conduct the interview.

### Data collection

Each participant is assigned a unique study number linked to the questionnaire and administered by study staff using a personal digital assistant (PDA). Study staff with training in phlebotomy will collect two micro-containers of whole blood for DBS and plasma specimens, whilst participants will be guided to collect a VVSS. Blood will be drawn from finger pricks, considered relatively non-invasive and not expected to negatively affect participation. Blood specimens will be stored in sterile containers and logged onto a laboratory tracking sheet. The specimens will then be couriered to a laboratory in KZN for testing. The blood is shipped daily from the study sites to the laboratory. The Standard Operating Procedure for taking blood is based on the protocols of the South African National Department of Health (NDoH) for taking blood for the HIV Polymerase chain reaction (PCR) test [45].

Study staff will administer a questionnaire to obtain:

- Socio-economic status of the household, including income, food security and location (urban or rural) and proximity to national roads, clinics and schools.
- Demographic information of study participants including age, gender, marital status, occupation, employment and educational status.
- Psycho-social information, including HIV knowledge, attitudes, perceived risk, self-efficacy and social norms related to HIV and AIDS, as well as depression indicators.
- Academic milestones of study participants, including academic progress and school completion.
- Behavioural information including age of sex initiation, partner characteristics (including age; number; type (regular/casual); circumcision status, HIV status); partner concurrency; condom use; engagement in transactional sex, substance (including alcohol) abuse and prevalence of intimate partner violence will be collected.
- Health seeking behaviour, including HIV testing (date of last HIV test, HIV test results) and HIV treatment (initiation date); STIs testing and treatment and contraceptive use.
- Exposure and engagement with DREAMS interventions.

### Access to health care

All study participants will be provided with a barcoded card and informed that they have an opportunity to access their HIV test results. Participants will be notified of the name of the clinic where their results will be sent. All participants’ laboratory HIV and pregnancy results will be sent to the nearest NDoH primary health clinic two weeks post collection of the biological samples for participants to access using their barcoded card. Participants will be encouraged to visit the clinic to obtain their results and receive appropriate counselling and referrals to access care and treatment.

HTS will be made available to study participants and household members wanting to know their HIV status immediately. They will be tested upon completion of data collection by the field worker using a rapid HIV test guided by SA NDoH HIV testing protocols [44]. Referrals to care, support and treatment will be made if required. Records of individuals referred for test results, treatment and care will be kept to monitor uptake of services.

### Outcomes and analysis

The primary outcome will be HIV incidence. The analysis will determine whether the aim of an HIV-incidence reduction of 40% at 2 years post implementation of the DREAMS programme in a ‘real world, non-trial setting’ was realised.

### Analysis of the primary objective

The study will use the Limiting antigen Avidity (LAg) Enzyme Immuno-Assay (EIA) to estimate recent HIV infection from the cross-sectional survey. The LAg EIA has been shown to be a reliable method of generating cross-sectional incidence estimates [46].

### Analysis of secondary objectives

**Measure HIV prevalence, and proportion on ART and ART naïve with detectable and undetectable HIV-1 viral load**: HIV prevalence will be expressed as the proportion testing HIV positive, where the denominator is all who provided a blood sample for HIV testing and had a conclusive result. The proportion of HIV positive AGYW on ART and those not on ART who have detectable or undetectable viral loads will be determined through laboratory tests that have a dynamic range of 20 to 10 million copies/ml. HIV-1 viral load data and Antiretroviral (ARV) drug measurement data will be used to determine the proportion of HIV infected persons on ART and ART naïve with detectable and undetectable viral loads. Participants who achieve viral suppression will be defined as those with a viral load < 1000 copies/ml. Estimated prevalence will take the survey design and sampling weights into account.

**Measure prevalence of pregnancies and sexually transmitted infections (STIs)**: Pregnancy and STIs (overall and individual diseases: (HBV, HSV-2, Syphilis, HPV, *C.trachomatis, N. gonorrhoea, T. vaginalis* and *M. genitalium*) will be calculated with 95% Confidence Interval (CIs). Pregnancy prevalence is defined as the proportion of AGYW testing positive for pregnancy from their blood samples, where all AGYW tested for pregnancy in this study is the denominator. Individual STI prevalence is calculated as the number of individuals testing positive for a particular STI, divided by all AGYW who were tested for STIs. Secondary analysis will be disaggregated by age, gender, marital status, occupation, employment status and educational attainment as well as other socioeconomic indicators and exposure to DREAMS interventions. All estimates will take the survey design and sampling weights into account.

**Measure risky sexual behaviour (e.g. number of [concurrent, lifetime, age-disparate] sexual partners, condom use, age of sexual debut), gender norms, intimate partner violence (including gender-based violence), and selected academic performance indicators (e.g. academic progress, school completion, assertive behaviour, participation in school and sport)**: The prevalence of risky sexual behaviour, gender norms and episodes of intimate partner violence (IPV) within the past year will be calculated with 95% CIs. IPV is assessed using behaviourally specific items developed by the World Health Organisation, and modified and used extensively in South Africa [47]. Five items ask about past experiences of physical violence from an intimate partner, whilst three items ask about sexual IPV in the previous year. Responses to each item are “never”, “once”, or “more than once”. A positive answer to any of the items leads to a woman being classified as having experienced IPV. Only women reporting having a boyfriend, husband, or having had sex with a man are asked these questions. Gender norms are measured using the gender equitable men (GEM) scale, this scale consists of 6 items relating to norms about male-female relationships and interactions. The scale asks respondents how much control their partners have over them in their relationships in relation to the clothes they wear, decisions made in the relationship and who they can spend time with outside of their relationship. Responses to each item are based on a Likert-type scale with responses ranging from strongly agree to strongly disagree. The scale items will be summed and a mean score calculated. Secondary analysis will be disaggregated by age, HIV and STI status and other socio-economic and academic indicators and exposure to DREAMS interventions.

**To measure the impact of economic empowerment, socio-economic status of their family, and selected parent/guardian/caregiver indicators have had on both the HIV incidence among AGYW and prevalence of GBV/IPV perpetrated against AGYW**: Composite scores will be created for AGYW using multiple correspondence analyses (MCA), taking the survey design and sampling weights into account.

**To measure the exposure to the DREAMS programme including condom promotion and provision (male and female); HTS and linkage to care; GBV prevention and post violence care and support; comprehensive sexual reproductive health rights (SRHR) services; social asset building interventions; school-based HIV and violence prevention; community mobilization and changes in social norms; parenting/caregiver programmes:** Composite scores will be created for AGYW and caregivers using self-reported data on their exposure to DREAMS activities. The mean score between AGYW exposed to DREAMS interventions and those who haven’t, will be compared using linear regression and by taking the survey design and sampling weights into account.

### Ethical considerations

Verbal consent (or assent) will be obtained from the head of the household for the household composition assessments. Consent will be obtained from all individuals 18 years and older and parental or guardian consent and individual assent will be obtained from all individuals who are younger than 18 years of age. Each study participant will be informed about the study and complete a consent form prior to enrolment, in accordance with 21 CFR Part 50 and ICH Good Clinical Practices (GCP) guidelines [48]. All consent forms and data collection instruments will be translated from English into the local languages. Back translations will be completed and reviewed by a bilingual independent source in order to ensure the accuracy of the translated information. Questionnaires will be administered in participants preferred language.

No personal identifiers will be documented on any of the data collection instruments. All participants will be assigned a unique study number (based on the number on the laboratory package). The study number will be linked to the questionnaire administered by study staff using a handheld PDA. Participants are required to provide written informed consent and assent for their biological samples to be stored beyond the conclusion of the study for any outstanding study related procedures, confirmation of results, HIV related testing and potential future testing. If participants do not consent to long-term storage and additional testing, their samples will be destroyed upon conclusion of the study and after completion of all listed protocol testing.

As the survey instrument contains questions on sensitive topics, including sexual behaviour, GBV, HIV status, access to care and treatment for HIV, it is possible that some individuals may experience discomfort during the interview process. Study staff will also be trained to identify and refer any AGYW to appropriate services who may be a victim of violence or a victim of trafficking, sexual abuse or exploitation, including those who self-identify as sex workers or report receiving money in exchange for sex.

As this is an observational study there are no anticipated adverse events. All unanticipated events will be documented and immediately reported to the Principle Investigator. These unanticipated events will be discussed and a verbal and/or written action plan will be devised and implemented within 48 hours of the submitted report. The study team will maintain written documentation on all events, including details of the action plan and event resolution. If necessary, a formal report will be sent to the Biomedical Research Ethics Committee (BREC) and reported to the Centers for Disease Control and Prevention (CDC) on the CDC’s Incident Report Form 1254 [45].

## Discussion

Ending the HIV/AIDS pandemic by 2030 requires the continual monitoring and evaluation of prevention programmes, with the aim of optimising efforts and ensuring the achievement of epidemic control. The monitoring of HIV prevalence is largely done through household surveys in SA [8]. However, key to determining the impact of initiatives targeting high-risk populations, including AGYW, is the collection of detailed data on the HIV response in a real world context, linking the scale-up of prevention efforts to changes in HIV incidence at a population level. This study will determine the impact DREAMS interventions have had on HIV incidence among AGYW in a ‘real world, non-trial setting’. This will be done by measuring HIV transmission within high-prevalence settings and within a high-risk population group, which will provide insights into the impact and efficacy of the DREAMS suite of prevention interventions.

The monitoring of temporal trends of HIV indicators will assist in improving and targeting prevention efforts and planning for health care delivery needs. This evaluation will be useful in implementing broader surveillance strategies and ensure they are supportive of the DREAMS interventions most effective to reduce risky sexual behaviour, increase knowledge of HIV status and subsequent uptake of ART, as well as reduce the prevalence of GBV.

This study has some limitations. Baseline incidence will need to be estimated. A longitudinal study design would have been preferred to establish baseline incidence prior to commencement of the DREAMS interventions and within the DREAMS targeted areas. Furthermore, a cross-sectional study cannot measure changes in behaviour and HIV incidence over time. This study would also not be able to determine the long-term impact of DREAMS being limited to information based on data gathered at a specific point in time. The study will only select participants from DREAMS targeted areas with the change in HIV infection rates estimated following the implementation of DREAMS programmes amongst AGYW through computation of the population’s attributable risk. As there is no non-DREAMS comparison arm in the study design, causal inferences regarding the relative contribution of DREAMS activities to measured reductions in key parameters is limited. The nature of the study did not allow for the establishment of a control group to assess the relative impact of the DREAMS interventions.

The approach of a population-based, cross-sectional survey to evaluate changes in HIV incidence post the DREAMS intervention implementation may limit the attribution of changes in HIV incidence to any specific programme intervention or combination of these. This evaluation will however, provide a reliable picture of HIV incidence amongst AGYW within the DREAMS targeted districts.

Data on intervention exposure, sexual behaviours, number of sexual partners and other sensitive issues will be self-reported and are thus subject to potential recall bias and social desirability bias. To limit the extent of the bias, field staff will be extensively trained on how to gain participant trust, manage sensitive information and assure confidentiality. In order to minimise familiarity, potentially leading to biased responses, fieldworkers will not be assigned to work in their own community.

The results of this study are expected to stand in contrast to findings from randomised controlled trials (RCTs). It is recognized that conclusions drawn from RCTs are not always useful in aiding health programme planning as they are applied to idealized environments, and are only able to measure efficacy in limited populations and fail to identify the complex environmental and community interactions within a study context [49, 50]. In contrast, non-trial data could provide valuable additional insights into the epidemiology of HIV within a real-world setting. Assessing the value of HIV interventions targeting AGYW, requires an evaluation of their impact within the current realities of this vulnerable population [51].

In summary, the highest HIV incidence rates in SA are amongst AGYW. DREAMS is a bold response to this high and sustained HIV-incidence among AGYW, with selected prevention interventions targeting this high-risk population with the aim of reducing HIV incidence by 40%. This study will measure the impact the implementation of DREAMS interventions have had on HIV incidence rates amongst AGYW and examine the factors that contribute to these changes amongst this population within selected districts in South Africa.

## Supplementary information

**Additional file 1.** STROBE Statement—checklist of items that should be included in reports of observational studies.

**Additional file 2.** Caregiver Questionnaire.

## Data Availability

Not applicable.

## Abbreviations

AGYW: Adolescent girls and young women
ANC: Antenatal care
ART: Antiretroviral therapy
ARV: Antiretroviral
BREC: Biomedical research ethics committee
CDC: Centers for disease control and prevention
CI: Confidence interval
CRSG: Community research support group
DBS: Dried blood spots
DREAMS: Determined resilient, empowered, aids-free, mentored and safe
EIA: Enzyme immuno-assa
FGD: Focus group discussion
FRR: False recent rate
GBV: Gender based violence
GCP: Good clinical practices
GP: Gauteng province
HBV: Hepatitis B virus
HIPSS: HIV Incidence provincial surveillance system
HPV: Human papillomavirus
HSV-2: Herpes simplex virus type 2
HTS: HIV Testing services
IE: Impact evaluation
KZN: KwaZulu-Natal
Lag: Limiting antigen avidity
MCA: Multiple correspondence analyses
NDoH: National department of health
OVC: Orphans and vulnerable children
PCR: Polymerase chain reaction
PDA: Personal data assistant
PEPFAR: President’s emergency plan for AIDS relief
PLHIV: People living with HIV
PMTCT: Prevention of mother-to-child transmission
PrEP: Pre-exposure prophylaxis
Py: Person years
SA: South Africa
SAL: Small area layers
SIMS: Site improvement and monitoring system
SRH: Sexual reproductive health
STI: Sexually transmitted infection
VVSS: Vulvo-vaginal swab sample
