# Supplementary material for: Evaluating DREAMS HIV prevention interventions targeting adolescent girls and young women in high HIV prevalence districts in South Africa: protocol for a cross-sectional study
Source: BMC Womens Health. 2020 Jan 16;20:7. doi: 10.1186/s12905-019-0875-2 (PMC6966796; doi:10.1186/s12905-019-0875-2)
Supplement: Supplementary file 2 — Additional file 2. Caregiver Questionnaire. [file 12905_2019_875_MOESM2_ESM.docx]

# Caregiver questionnaire

Caregiver Questionnaire

Study Title: DREAMS impact evaluation -

HIV incidence trends among adolescent girls and young women in the eThekwini (KZN), uMgungundlovu (KZN), City of Johannesburg (GP) and Ekurhuleni (GP) districts, South

Africa

**Version 7.0 20 December 2016**

Since you have agreed to participate in the DREAMS Implementation Evaluation Survey, we would like to ask you a few questions that will help us better understand the impact of the DREAMS programme.

Please feel free to ask any questions that you might have about what we would be asking you.

We will focus on questions on:

- Generally about yourself, your family and your environment.
- Your parenting practices.
- Your exposure to DREAMS activities.

We hope that you would answer all the questions. Please note that your name will not appear anywhere on the questionnaire, your answers will be kept private and no one will know that the answers came from you. We hope that you answer the questions truthfully and to the best of your ability. Once again please feel free to ask any questions you might feel are important to know.

|  | **Section: Pre program mobile database** |
| --- | --- |
|  | Staff ID  Household ID number  GPS Co-ordinates  Location  Map  Date |
|  | **Section: Participant Criteria** |
|  | **Eligibility**  **Not eligible if answered yes to any of the following questions:**   - Adolescent linked to caregiver younger than 12 or 18 years of age or older. - Non-resident from the study area. - Refusal by caregiver (parent/guardian) to participate in the study. (No consent.) |
|  | **Refusal**   - **What are the reasons that you do not want to participate?** - Caregiver (parent/guardian) declined to give reason (s) for refusal. - I don’t have time to participate in the survey. - Adolescent refused - I find the topics uncomfortable or embarrassing - I don’t want my adolescent’s samples taken - Other _______________ |
|  | **Section: Demographics** |
|  | **How old were you at your last birthday? ^^[[1]](#footnote-1)^^**   - (Age of the respondent) |
|  | **What is your highest school qualification achieved? ^^[[2]](#footnote-2)^^**   - Grade R - Sub a/class 1/grade 1 - Sub b/class 2/grade 2 - Standard 1/grade 3 /ABET 1 - Standard 2/grade 4 / ABET 2 - Standard 3/grade 5/ABET 2 - Standard 4/grade 6/ABET 3 - Standard 5/grade 7/ABET 3 - Standard 6/grade 8/ABET 3 - Standard 7/grade 9/ABET 3 - Standard 8/grade 10/NTC 1 - Standard 9/grade 11/NTC 2 - Standard 10/grade 12/NTC 3 - Further studies incomplete - Diploma/other post school completed - Further degree completed - Don’t know - No schooling |
|  | **What is your home language?**   - Zulu - Xhosa - Sotho - Tswana - English - Afrikaans - Other ______________. |
|  | **Race of the respondent^[[3]](#footnote-3)^**   - African - White - Coloured - Indian/Asian - Other |
|  | **What is your nationality?^[[4]](#footnote-4)^**   - South African citizen - Non-citizen (Permanent resident ) - Non-citizen (Refugee) - Other |
|  | **What is your current relationship or marital status?^[[5]](#footnote-5)^**   - Married (living with husband/wife) - Married (not living with husband/wife) - Living together, not married (living with boyfriend/girlfriend/partner) - Going steady (in a relationship, but not living together) - Single (not in a relationship) - Divorced / separated - Widower / Widow - Civil Union - Other |
|  | **What is your relationship to the adolescent?**   - Mother/Father - Maternal Grandparent (Grandparent from the mothers side) - Paternal Grandparent (Grandparent from the fathers side) - Aunt/Uncle - Cousin - Other family - Not blood related |
|  | **If caregiver is not the biological mother:**  **Is the biological mother still alive**?   - No - Yes - Don't know - Refused |
|  | **Does the biological mother live in the same house as the adolescent?**   - Always - Sometimes - Never - Refused |
|  | **If caregiver is not the biological father:**  **Is the biological father still alive?**   - No - Yes - Don't Know - Refused |
|  | **Does the biological father live in the same house as the adolescent?**   - Always - Sometimes - Never - Refused |
|  | **Household composition** |
|  | **How many people usually live in your household (including yourself)**   - **___**Number |
|  | **18 years and older**   - **___**_ Number of men - ____ Number of women |
|  | **Younger than 18**   - **____** Number of Boys - ____Number of Girls |
|  | **How many people in your household have died in the last year? Please provide numbers**   - **___**Number |
|  | **18 years and older**   - **___**_ Number of men - ____ Number of women |
|  | **Younger than 18**   - **____** Number of Boys - ____Number of Girls |
|  | **Household economic status** |
|  | **What is the main source of water for members of your Household?**   - Pipe water (tap) in dwelling - Pipe water (tap) in yard - Bottled water - Water tank’ - Rain water tank (Jo-Jo tank) - Borehole, well or spring - Dam/river or stream - Public / communal tap - Other |
|  | **What kind of toilet facilities does your household have?**   - Flushing toilet (own) - Flushing toilet (shared) - Bucket laterine - Pit latrine with ventilation - Pit latrine without ventilation - No toilet / bush / field - Other |
|  | **What is this households main source of energy?**   - Electricity - Coal - Wood - Gas - Parafin - Animal dung - Other |
|  | **Does your household have any of the following in working condition?**   - **Electricity** - **Radio** - **Televison** - **Landline phone** - **Cell phone** - **Refridgerator** - **Personal computer** - **Washing machine** |
|  | **Did this household receive any income this month?**   - No - Yes |
|  | **What is the main form of income of this household ?**   - Formal salary/wages where tax is paid - Salary/earnings where no tax is paid - Adult Family members or relatives help contribute - Younger family members or relatives help contribute - Government grants - Grants/donations by private welfares - Other__________________________ |
|  | **What is this household total monthly income?**   - **_____** |
|  | **Does this household receive any**   - Child support grants - Care dependency grant - Foster child grant |
|  | **Has the household become wealthier , poorer or stayed the same?**   - Wealthier - Poorer - Stayed the same |
|  | **In the past 4 weeks how often was there no food to eat of any kind in your house because of lack of money?**   - Often - Sometimes - Rarely - Never |
|  | **In the past 4 weeks , how often did you or any member of your household go to sleep hungry because of lack of food?**   - Often - Sometimes - Rarely - Never |
|  | **In the past 4 weeks , how often did you or any of your household go to a whole day and night without eating because of lack of food?**   - Often - Sometimes - Rarely - Never |
|  | **Caregiver Explanatory Variables** |
|  | **Physical health**  **At any point in the last month, have you gone more than one day when you were too sick or too tired to participate in daily activities?^[[6]](#footnote-6)^**   - No - Yes |
|  | **How often did it happen in the last month that you were too sick or too tired to participate in daily activities would you say?**   - Less than once a week - At least once a week - More than once a week - No response |
|  | **In the past 12 months, have you had an illness or serious medical condition for 3 months in a row or longer?**   - No - Yes - Don’t Know |
|  | **In the past 12 months, have any adults who still live in the household EXCLUDING yourself had an illness for 3 months in a row or longer?**   - No - Yes - Don’t Know |
|  | **Have you ever had an HIV test?**   - Yes - No |
|  | **How long ago did you have your most recent HIV test?**   - 0 to 6 months - 7 to 12 months - Between 1-2 years ago - Between 2-3 years ago - Three or more years ago |
|  | **Is any of the adults living in your household HIV positive?**   - No - Yes - Don’t Know |
|  | **What was the result of your test?**   - HIV negative - HIV Positive - Refused to disclose - Indeterminate |
|  | **Do you have difficulty seeing, even if wearing glasses?^[[7]](#footnote-7)^**   - No difficulty - Yes – some difficulty - Yes – a lot of difficulty - Cannot do at all |
|  | **Do you have difficulty hearing, even if using a hearing aid?**   - No difficulty - Yes – some difficulty - Yes – a lot of difficulty - Cannot do at all |
|  | **Do you have difficulty walking or climbing steps?**   - No difficulty - Yes – some difficulty - Yes – a lot of difficulty - Cannot do at all |
|  | **Do you have difficulty remembering or concentrating?**   - No difficulty - Yes – some difficulty - Yes – a lot of difficulty - Cannot do at all |
|  | **Do you have difficulty remembering or concentrating?**   - No difficulty - Yes – some difficulty - Yes – a lot of difficulty - Cannot do at all |
|  | **Do you have difficulty speaking?**   - No difficulty - Yes – some difficulty - Yes – a lot of difficulty - Cannot do at all |
|  | **Psychological health** |
|  | **We would like you to describe way that you may have felt or behaved during the last week:^[[8]](#footnote-8)^**  **I felt depressed**   - Rarely (Less than 1 day) - Some of the time (1-2 days) - Occasionally (3-4 days) - All of the time (5-7 days) |
|  | **My sleep was restless**   - Rarely (Less than 1 day) - Some of the time (1-2 days) - Occasionally (3-4 days) - All of the time (5-7 days) |
|  | **I had crying spells**   - Rarely (Less than 1 day) - Some of the time (1-2 days) - Occasionally (3-4 days) - All of the time (5-7 days) |
|  | **I felt lonely**   - Rarely (Less than 1 day) - Some of the time (1-2 days) - Occasionally (3-4 days) - All of the time (5-7 days) |
|  | **I could not get going**   - Rarely (Less than 1 day) - Some of the time (1-2 days) - Occasionally (3-4 days) - All of the time (5-7 days) |
|  | **Parenting Practices** |
|  | Should be asked per adolescent: Adolescent 001, 002  **I have pleasant conversations with [Adolescent Name].^[[9]](#footnote-9)^**   - Never - Almost never - Sometimes - Often - Always |
|  | **I try to teach [Adolescent Name] new things.**   - Never - Almost never - Sometimes - Often - Always |
|  | **[Adolescent Name] and I hug and/or kiss each other.**   - Never - Almost never - Sometimes - Often - Always |
|  | **I have pleasant conversations with [Adolescent Name].^[[10]](#footnote-10)^**   - Never - Almost never - Sometimes - Often - Always |
|  | **I try to teach [Adolescent Name] new things.**   - Never - Almost never - Sometimes - Often - Always |
|  | **[Adolescent Name] and I hug and/or kiss each other.**   - Never - Almost never - Sometimes - Often - Always |
|  | **I laugh with [Adolescent Name] about things we find funny.**   - Never - Almost never - Sometimes - Often - Always |
|  | **[Adolescent Name] and I spend time together doing fun activities**   - Never - Almost never - Sometimes - Often - Always |
|  | **I listen to [Adolescent Name]'s feelings and try to understand them.**   - Never - Almost never - Sometimes - Often - Always |
|  | **I thank or praise [Adolescent Name].**   - Never - Almost never - Sometimes - Often - Always |
|  | **I offer to help, or help [Adolescent Name] with things she is doing.**   - Never - Almost never - Sometimes - Often - Always |
|  | **I comfort [Adolescent Name] when she seems scared, upset, or unsure.**   - Never - Almost never - Sometimes - Often - Always |
|  | **I hold or touch [Adolescent Name] in an affectionate way.**   - Never - Almost never - Sometimes - Often - Always |
|  | **I ask [Adolescent name] about her day.**   - Never - Almost never - Sometimes - Often - Always |
|  | **Support parent** |
|  | **How much you would say you really know about each of the following situations: ^[[11]](#footnote-11)^**  **Where [Adolescent name] goes at night?**   - Do Not Know - Sometimes Know - Always Know |
|  | **What [Adolescent name] does with their free time?**   - Do Not Know - Sometimes Know - Always Know |
|  | **Who [Adolescent name] friends are?**   - Do Not Know - Sometimes Know - Always Know |
|  | **Talking about sex** |
|  | **I would be embarrassed talking to my child about sex**   - Disagree - Agree - Neutral |
|  | **I would NOT answer my child’s questions about sex**   - Disagree - Agree - Neutral |
|  | **I do not talk to my child about sex, because she knows about it.**   - Disagree - Agree - Neutral |
|  | **My child is too young for me to be able to talk about sex.**   - Disagree - Agree - Neutral |
|  | **If I thought my child was sexually active , I would overlook it.**   - Disagree - Agree - Neutral |
|  | **If my child had a blessor/sugar daddy , I would accept it.**   - Disagree - Agree - Neutral |
|  | **Adolescent Explanatory Variables^[[12]](#footnote-12)^** |
|  | **I am going to read some statements that describe adolescent behaviour. For each one, please indicate how much this statement describes each of [AdolescentName]’s behaviour now or in the past four weeks. The responses are "NOT TRUE", "SOMEWHAT TRUE", or "VERY TRUE".**  **Acts too young for her age**   - Not true - Somewhat true - Very true |
|  | **Argues a lot**   - Not true - Somewhat true - Very true |
|  | **Fails to finish things she starts**   - Not true - Somewhat true - Very true |
|  | **Can't concentrate, can't pay attention for long**   - Not true - Somewhat true - Very true |
|  | **Can't sit still, restless, or hyperactive**   - Not true - Somewhat true - Very true |
|  | **Destroys things belonging to her family or others**   - Not true - Somewhat true - Very true |
|  | **Disobedient at home**   - Not true - Somewhat true - Very true |
|  | **Disobedient at school**   - Not true - Somewhat true - Very true |
|  | **Feels worthless or inferior**   - Not true - Somewhat true - Very true |
|  | **Impulsive or acts without thinking**   - Not true - Somewhat true - Very true |
|  | **Too fearful or anxious**   - Not true - Somewhat true - Very true |
|  | **Feels too guilty**   - Not true - Somewhat true - Very true |
|  | **Self-conscious or easily embarrassed**   - Not true - Somewhat true - Very true |
|  | **Inattentive or easily distracted**   - Not true - Somewhat true - Very true |
|  | **Stubborn, sullen, or irritable**   - Not true - Somewhat true - Very true |
|  | **Temper tantrums or hot temper**   - Not true - Somewhat true - Very true |
|  | **Threatens people**   - Not true - Somewhat true - Very true |
|  | **Unhappy, sad, or depressed**   - Not true - Somewhat true - Very true |
|  | **Worries**   - Not true - Somewhat true - Very true |
|  |  |
|  |  |
|  | **Discipline** |
|  | **How often did you shout, yell, or scream at [AdolescentName]?**   - Never - has occurred but not in the past 4 weeks - once or twice - three to five times - more than 5 times |
|  | **How often did you say you would send him/her away or kick him/her out of the house?**   - Never - has occurred but not in the past 4 weeks - once or twice - three to five times - more than 5 times |
|  | **How often did you call [AdolescentName] “dumb” or “lazy” or some other name like that?**   - Never - has occurred but not in the past 4 weeks - once or twice - three to five times - more than 5 times |
|  | **How often did you smack [AdolescentName] using your hand?**   - Never - has occurred but not in the past 4 weeks - once or twice - three to five times - more than 5 times |
|  | **How often did you hit [AdolescentName] with a strap, belt, or similar object?**   - Never - has occurred but not in the past 4 weeks - once or twice - three to five times - more than 5 times |
|  | **Exposure to dreams** |
|  | **In the last 12 months has any adolescent in your care received cash transfer or educational subsidy (excluding child support / care dependency / foster child grants)? to help them stay in school?**   - No - Yes - Not sure |
|  | **In the last 12 months have you received any training related to parenting or providing care to the children in your household?**   - No - Yes - Not sure |
|  | **In the last 12 months have you received training/education on the benefit of HIV testing and getting to know your status?**   - No - Yes - Not sure |

# Cross-Sectional Study Questionnaire (18+ years)

**AGYW Questionnaire**

**18 to 24 years of age**

**Study Title: DREAMS impact evaluation -**

**HIV incidence trends among adolescent girls and young women in the eThekwini (KZN), uMgungundlovu (KZN), City of Johannesburg (GP) and Ekurhuleni (GP) districts, South Africa**

**Version 7.0 20 December 2016**

Since you have agreed to participate in the DREAMS Implementation Evaluation Survey, we would like to ask you a few questions that will help us better understand the impact of the DREAMS programme.

Please feel free to ask any questions that you might have about what we would be asking you. We will focus on questions on:

- Generally about yourself, your family and your environment
- Some of your feelings, thoughts and hopes for the future
- Some behaviours including sexual behaviours that might predispose you to HIV
- Where you access health care
- About HIV and AIDS

We hope that you would answer all the questions. Please note that your name will not appear anywhere on the questionnaire, your answers will be kept private and no one will know that the answers came from you. We hope that you answer the questions truthfully and to the best of your ability. Once again please feel free to ask any questions you might feel are important to know.

|  | **Section: Pre program mobile database** |
| --- | --- |
|  | Staff ID  Household ID number  GPS Co-ordinates  Location  Map  Date |
|  | **Section: Participant Criteria** |
|  | **Eligibility**  **Not eligible if answered yes to any of the following questions:**   - Younger than 12 or older than 24 years of age. - Non-resident from the study area. - Refusal by participant to participate in the study. (No consent.) - Refusal by participant to provide clinical samples of DBS/peripheral blood and self-collected vulvo-vaginal swab samples. - Cognitive or mental challenges (based on the assessment of the participant’s ability to comprehend the study information provided). - Deaf or mute. - Not speaking English, IsiZulu, Sotho, Afrikaans. |
|  | **2nd/3rd survey:**   - Have not been living in the area in the past year (2nd survey)/past 2 years (3rd survey). |
|  | **Refusal**  **What are the reasons that you do not want to participate?**   - Participant declined to give a reason(s) for refusal - I don’t have time to participate in the survey - I already know I am HIV positive - I don’t wish to be retested for HIV - I find the topics uncomfortable or embarrassing - I don’t want my samples taken - Other (specify) _______________ |
|  | **Consent**  **Age of participant**   - ≥18 years |
|  | **Consent for study participation**   - Individual consent only |
|  | **Consent for sample storage and future testing**   - Individual consent only |
|  | **Sample collection**  **Barcode**   - Scan Barcode |
|  | **Sample collection:**   - Blood sample - Self collected Vulvo-vaginal swabs |
|  | **Demographics** |
|  | **How old were you at your last birthday? ^^[[13]](#footnote-13)^^**   - (Age of the respondent) |
|  | **Information**  **What is your home language?**   - Zulu - Xhosa - Sotho - Tswana - English - Afrikaans - Other ______________. |
|  | **Race of the respondent**   - African - White - Coloured - Indian/Asian - Other |
|  | **What is your nationality?**   - South African citizen - Non-citizen (Permanent resident ) - Non-citizen (Refugee) - Other |
|  | **Mobility**  **How long have you lived in this community?**   - > 5 years - 1-5 years - <1 year |
|  | **In the past 12 months have you been away from your usual residence for more than one month?**   - Yes - No |
|  | **In the past week, how many nights have you stayed away from home?**   - ______ Number of days |
|  | **Is your biological mother still alive**?   - No - Yes - Don't know - Refused |
|  | **Does your biological mother live in the same house as you ?**   - Always - Sometimes - Never - Refused |
|  | **Is your biological father still alive?**   - No - Yes - Don't Know - Refused |
|  | - **Does your biological father live in the same house as you ?**Always - Sometimes - Never - Refused |
|  | **Household composition** |
|  | **How many people usually live in your household (including yourself)**   - **___**Number |
|  | **18 years and older**   - **___**_ Number of men - ____ Number of women |
|  | **Younger than 18**   - **____** Number of Boys - ____Number of Girls |
|  | **How many people in your household have died in the last year? Please provide numbers**   - **___**Number |
|  | **Household economic status** |
|  | **What is the main source of water for members of your Household?**   - Pipe water (tap) in dwelling - Pipe water (tap) in yard - Bottled water - Water tank’ - Rain water tank (Jo-Jo tank) - Borehole, well or spring - Dam/river or stream - Public / communal tap - Other |
|  | **What kind of toilet facilities does your household have?**   - Flushing toilet (own) - Flushing toilet (shared) - Bucket laterine - Pit latrine with ventilation - Pit latrine without ventilation - No toilet / bush / field - Other |
|  | **What is this households main source of energy?**   - Electricity - Coal - Wood - Gas - Paraffin - Animal dung - Other |
|  | **Does your household have any of the following in working condition?**   - Electricity - Radio - Television - Landline phone - Cell phone - Refrigerator - Personal computer - Washing machine |
|  | **Did this household receive any income this month?**   - No - Yes |
|  | **What is the main form of income of this household ?**   - Formal salary/wages where tax is paid - Salary/earnings where no tax is paid - Adult Family members or relatives help contribute - Younger family members or relatives help contribute - Government grants - Grants/donations by private welfares - Other__________________________ |
|  | **What is this household total monthly income?**   - **_____** |
|  | **Does this household receive any**   - Child support grants - Care dependency grant  (Grant given to parents with children who have mental or physical disabilities) - Foster child grant |
|  | **Has the household become wealthier, poorer or stayed the same?**   - Wealthier - Poorer - Stayed the same |
|  | **In the past 4 weeks how often was there no food to eat of any kind in your house because of lack of money?**   - Often - Sometimes - Rarely - Never |
|  | **In the past 4 weeks , how often did you or any member of your household go to sleep hungry because of lack of food?**   - Often - Sometimes - Rarely - Never |
|  | **In the past 4 weeks , how often did you or any of your household go to a whole day and night without eating because of lack of food?**   - Often - Sometimes - Rarely - Never |
|  | **Education** |
|  | **Do you currently attend school?**   - Yes - No |
|  | **What is your highest school qualification achieved?**   - Grade R - Sub a/class 1/grade 1 - Sub b/class 2/grade 2 - Standard 1/grade 3 /ABET 1 - Standard 2/grade 4 / ABET 2 - Standard 3/grade 5/ABET 2 - Standard 4/grade 6/ABET 3 - Standard 5/grade 7/ABET 3 - Standard 6/grade 8/ABET 3 - Standard 7/grade 9/ABET 3 - Standard 8/grade 10/NTC 1 - Standard 9/grade 11/NTC 2 - Standard 10/grade 12/NTC 3 - Further studies incomplete (skip to employment) - Diploma/other post school completed (skip to employment) - Further degree completed (skip to employment) - Don’t know - No schooling (skip to employment) |
|  | **If not: Why not?**   - My family does not have enough money - I don’t like school - I have to look after my younger brothers/sisters - I have to look after a sick family member - I failed - I was expelled - I became pregnant (if female) - Poor health - Completed grade 12 (skip to employment) - Other |
|  | **If yes: Are you repeating this grade?**   - Yes - No |
|  | **If yes: Why?**   - My family did not have enough money - I don’t like school - I had to look after my younger brothers/sisters - I had to look after a sick family member - I failed - I was expelled - I was pregnant / had a baby / had a child (if female) - I had been sick - Other |
|  | **If attending school: In the last month, have you missed any days of school? (excluding holidays)^^[[14]](#footnote-14)^^**   - Yes: How many days? - No |
|  | **If yes: Why have you missed school?^^[[15]](#footnote-15)^^**   - I have been sick - I don’t feel safe going to school - I don’t feel safe at school - I don’t like school food - I have to look after my younger brothers/sisters - I have to look after a sick family member - I don’t have enough money to go to school everyday - Exams were done - I am pregnant/have a baby/have a child - Other |
|  | **If yes, Family members/carers think that performing well in school is very important^^[[16]](#footnote-16)^^**   - Strongly disagree - Disagree - I don’t agree or disagree - Agree - Strongly agree |
|  | **Employment** |
|  | **How would you describe your present employment situation?**   - Housewife, homemaker, not looking for work - Housewife, homemaker, looking for work - Unemployed, looking for work - Unemployed, not looking for work - Work in informal sector, looking for permanent work - Work in informal sector, not looking for permanent work - Sick/disabled and unable to work - Student/pupil/learner - Self-employed - full time (40 hours or more per week) - Self-employed - part time (less than 40 hours per week) - Employed part time (if none of the above) (less than 40 hours per week) - Employed full time (40 hours or more per week) - Other |
|  | **If employed, considering all the money you earned from jobs or selling things, how much did you earn in the last 4 weeks (not including grants)?**   - Numeric response |
|  | **Do you save money? For example, putting some money aside for another day or for a special purpose?**   - Yes: How much do you have in savings? R - No - Refused |
|  | **If in school: Do you receive money from a program to stay in school?**   - Yes, total amount R - No - Refused |
|  | **Health** |
|  | **At any time in the last two weeks, have you been too sick to participate in daily activities? ^^[[17]](#footnote-17)^^**   - Yes - No |
|  | **Do you have difficulty seeing, even if wearing glasses?^^[[18]](#footnote-18)^^**   - No difficulty - Yes – some difficulty - Yes – a lot of difficulty - Cannot do at all |
|  | **Do you have difficulty hearing, even if using a hearing aid?**   - No difficulty - Yes – some difficulty - Yes – a lot of difficulty - Cannot do at all |
|  | **Do you have difficulty walking or climbing steps?**   - No difficulty - Yes – some difficulty - Yes – a lot of difficulty - Cannot do at all |
|  | **Do you have difficulty remembering or concentrating?**   - No difficulty - Yes – some difficulty - Yes – a lot of difficulty - Cannot do at all |
|  | **Do you have difficulty remembering or concentrating?**   - No difficulty - Yes – some difficulty - Yes – a lot of difficulty - Cannot do at all |
|  | **Do you have difficulty speaking?**   - No difficulty - Yes – some difficulty - Yes – a lot of difficulty - Cannot do at all |
|  | **Psychological health** |
|  | **We would like you to describe way that you may have felt or behaved during the last week:^^[[19]](#footnote-19)^^**  **I felt depressed**   - Rarely (Less than 1 day) - Some of the time (1-2 days) - Occasionally (3-4 days) - All of the time (5-7 days) |
|  | **My sleep was restless**   - Rarely (Less than 1 day) - Some of the time (1-2 days) - Occasionally (3-4 days) - All of the time (5-7 days) |
|  | **I had crying spells**   - Rarely (Less than 1 day) - Some of the time (1-2 days) - Occasionally (3-4 days) - All of the time (5-7 days) |
|  | **I felt lonely**   - Rarely (Less than 1 day) - Some of the time (1-2 days) - Occasionally (3-4 days) - All of the time (5-7 days) |
|  | **I could not get going**   - Rarely (Less than 1 day) - Some of the time (1-2 days) - Occasionally (3-4 days) - All of the time (5-7 days) |
|  | **Resilience** |
|  | **I am able to solve problems without harming myself or others**.^^[[20]](#footnote-20)^^   - Not at all - A little - Somewhat - Quite a bit - A lot |
|  | **I know where to go in the community to get help.**   - Not at all - A little - Somewhat - Quite a bit - A lot |
|  | **Getting an education is important to me.**   - Not at all - A little - Somewhat - Quite a bit - A lot |
|  | **I try to finish what I start.**   - Not at all - A little - Somewhat - Quite a bit - A lot |
|  | **I have people I look up to.**   - Not at all - A little - Somewhat - Quite a bit - A lot |
|  | **My parents/caregivers know a lot about me.**   - Not at all - A little - Somewhat - Quite a bit - A lot |
|  | **My family stands by me during difficult times.**   - Not at all - A little - Somewhat - Quite a bit - A lot |
|  | **My friends stand by me during difficult times.**   - Not at all - A little - Somewhat - Quite a bit - A lot |
|  | **I have opportunities to develop skills that will be useful later in life.**   - Not at all - A little - Somewhat - Quite a bit - A lot |
|  | **I am treated fairly in my community.**   - Not at all - A little - Somewhat - Quite a bit - A lot |
|  | **I feel I belong in my / community**   - Not at all - A little - Somewhat - Quite a bit - A lot |
|  | **I enjoy my cultural and family traditions.**   - Not at all - A little - Somewhat - Quite a bit - A lot |
|  | **Caregiver Relationships** |
|  | **We are now going to ask you some questions about your caregiver. By caregiver, we mean a parent, legal guardian or other adult who is primarily responsible for your well-being; specifically, the person that prepared meals sought medical attention when you were ill and otherwise cared for you while you were growing up.**  **Who would you say was your primary caregiver currently?**   - Mother - Father - Grandmother - Grandfather - Aunt - Uncle - Sister - Brother - Other - Relative - Non-relative |
|  | ***The next set of questions asks you about your relationship with your caregiver. Remember that your answers are confidential and we will not share them with your caregiver. For each statement, please tell us how true it is for you. The responses are "NOT AT ALL TRUE" "A LITTLE TRUE" "MOSTLY TRUE" OR "COMPLETELY TRUE"^^[[21]](#footnote-21)^^***  **Your [Caregiver] respected your feelings.**   - Not at all true - A little true - Mostly true - Completely true |
|  | **You feel your [Caregiver] did a good job as a caregiver.**   - Not at all true - A little true - Mostly true - Completely true |
|  | **Your [Caregiver] accepted you as you are.**   - Not at all true - A little true - Mostly true - Completely true |
|  | **You liked to get your [Caregiver]’s point of view on things you were concerned about.**   - Not at all true - A little true - Mostly true - Completely true |
|  | **Your [Caregiver] could tell when you were upset about something.**   - Not at all true - A little true - Mostly true - Completely true |
|  | **When you discussed things, your [Caregiver] cared about your point of view.**   - Not at all true - A little true - Mostly true - Completely true |
|  | **Your [Caregiver] trusted your judgment.**   - Not at all true - A little true - Mostly true - Completely true |
|  | **Your [Caregiver] helped you to understand yourself better.**   - Not at all true - A little true - Mostly true - Completely true |
|  | **You told your [Caregiver] about your problems and troubles**   - Not at all true - A little true - Mostly true - Completely true |
|  | **Your [Caregiver] helped you to talk about your difficulties**   - Not at all true - A little true - Mostly true - Completely true |
|  | **Your [Caregiver] understood you.**   - Not at all true - A little true - Mostly true - Completely true |
|  | **When you were angry about something, your [Caregiver] tried to be understanding.**   - Not at all true - A little true - Mostly true - Completely true |
|  | **You trusted your [Caregiver].**   - Not at all true - A little true - Mostly true - Completely true |
|  | **You could count on your [Caregiver] when you needed to get something off your chest.**   - Not at all true - A little true - Mostly true - Completely true |
|  | **If your [Caregiver] knew something was bothering you, they asked you about it.**   - Not at all true - A little true - Mostly true - Completely true |
|  | **Have you spoken to your caregiver in the last 12 months?**   - No (Skip to next section) - Yes |
|  | **In the last 12 months, how often did your caregiver talk to you about:**  **Unhealthy friendships or relationships?**   - Not at all - Only a little; - Quite a lot - Great deal |
|  | **Talked about HIV/AIDS?**   - Not at all - Only a little; - Quite a lot - Great deal |
|  | **Talked about sex?**   - Not at all - Only a little; - Quite a lot - Great deal |
|  | **Talked about condoms?**   - Not at all - Only a little; - Quite a lot - Great deal |
|  | **Talked about sexually transmitted diseases, or STDs?**   - Not at all - Only a little; - Quite a lot - Great deal |
|  | **Family planning** |
|  | **Are you currently on any contraceptive method?^^[[22]](#footnote-22)^^**   - Yes - No - Refused |
|  | **If no, why not?**   - Trying to have a baby - Cannot access contraceptive methods - Have not thought about getting on it. - Not having sex/abstaining - Currently pregnant - Scared - I am too young - Worried about contraceptive side effects/Experienced contraceptive side effects in the past - Other |
|  | **If yes, Which form(s) of contraceptives are you using?**   - Condoms - Depo-provera (3 month injection) - Nur-isterate (2 month injection) - Oral Pills taken daily - Implant - Intrauterine contraceptive device (IUCD) - Other, specify________ |
|  | **Have you ever been pregnant?**   - Yes - No - Refused |
|  | **If yes, How many times have you been pregnant?**   - [number] |
|  | **If yes, What was your age at your first pregnancy?**   - [number] |
|  | **If yes, were you still at school when you were pregnant?**   - Yes - No - Refused   **If yes, Did you/will you return to school afterwards?**   - Yes - No - Refused |
|  | **If yes, the last time you became pregnant did you:**   - Want to become pregnant then - Want to wait until later to become pregnant - Not want to have any children |
|  | **If yes, what was the outcome of your pregnancy? (each pregnancy)**   - Had termination of pregnancy (TOP) by unqualified person (backdoor abortion). - Had termination of pregnancy (TOP) by clinic staff. - Lost naturally (spontaneously abortion). - Still pregnant. - Live child/ children. |
|  | **If yes, in case of live child/children:**  **Do you receive a child support grant? (per child)**   - Yes - No - Refused |
|  | **Knowledge & perceptions of HIV** |
|  | **Can people reduce their chances of getting HIV (the virus that causes AIDS) by having just one sex partner who is not infected and who has no other partners?’^^[[23]](#footnote-23)^^**   - Yes - No - Don’t know |
|  | **Can people reduce their chances of getting HIV/AIDS by using a condom every time they have sex?**   - Yes - No - Don’t know |
|  | **Can a person get HIV/AIDS from mosquito bites?**   - Yes - No - Don’t know |
|  | **Can people get HIV by sharing food with a person who has HIV/AIDS?**   - Yes - No - Don’t know |
|  | **Is it possible for a healthy-looking person to have HIV/AIDS?**   - Yes - No - Don’t know |
|  | **Can having sex with a virgin cure AIDS?**   - Yes - No - Don’t know |
|  | **Can people get HIV or AIDS because of witchcraft or other supernatural means?**   - Yes - No - Don’t know |
|  | **Have you ever heard of a treatment for HIV – the virus that causes AIDS – called Antiretroviral Therapy (ART)**   - Yes - No - Don’t know |
|  | **If yes: If HIV positive people take Antiretroviral Therapy (ART) can this reduce their risk of transmitting HIV to another person?**   - Yes - No - Don’t know   **Have you ever heard of Pre-exposure Prophylaxis (PrEP)?**   - Yes - No - Don’t know |
|  | **Is a woman's risk of getting HIV reduced if her partner is circumcised?**   - Yes - No - Don’t know |
|  | **Is a woman completely protected from HIV infection if her partner is circumcised?**   - Yes - No - Don’t know |
|  | **HIV status** |
|  | **Do you know of a place nearby where you can get an HIV test?**   - Yes - No |
|  | **Have you ever had an HIV test?**   - Yes - No |
|  | **If yes, How long ago did you have your most recent HIV test?**   - 0 to 6 months - 7 to 12 months - Between 1-2 years ago - Between 2-3 years ago - Three or more years ago |
|  | **If tested in the past 12 months:**  **Where did you get tested?**   - School/university/FET campaign - Community testing campaign - Clinic/hospital - Other |
|  | **If tested in the past 12 months:**  **Did you test together with a partner in the past 12 months?**   - Yes - No |
|  | **Have you been told/informed of the result of your most recent HIV test?**   - Yes - No |
|  | **If never had a test: What are your reasons for not having ever had an HIV test?(multiple answers)**   - I do not know where to get tested - I do not think that I have HIV - I am not at risk for HIV - I trust my partner - I was afraid to find out that I might be HIV positive - I am not ready to have an HIV test - I was concerned about CONFIDENTIALITY - I was concerned about STIGMA, DISCRIMINATION or REJECTION - I was concerned about LOSING MY JOB - I am concerned about the STANDARD OF SERVICE - I haven’t got around to it - Other |
|  | **If ever had a test: What was the result of your latest HIV test (if indicated received)?**   - HIV negative. - HIV positive. - Indeterminate. - Refused. |
|  | **HIV positive only (Skip if HIV negative, indeterminate or refused)** |
|  | **Not including medical personnel or those at the clinic, have you talked to anyone about your HIV status?**   - Nobody - Yes, One person - Yes, More than one person |
|  | **Are you currently being provided with any of the following support?**   - Nutritional support - Emotional support - Treatment buddy - Home based care - Financial support - Treatment of opportunistic infections |
|  | **Have you ever been pregnant while you were HIV positive? (Skip to 130 if never been pregnant )**   - Yes - No |
|  | **If yes, Did your baby become infected with HIV?**   - Yes - No |
|  | **Did you take ART while pregnant to reduce the chances of your child acquiring HIV?**   - Yes - No |
|  | **Risk perceptions** |
|  | **I feel that the chances are high that I can get HIV [skip if already HIV positive]**   - Strongly Agree - Agree - Disagree - Strongly Disagree |
|  | **I believe that older men are more likely to have HIV than younger men.**   - Strongly Agree - Agree - Disagree - Strongly Disagree |
|  | **I feel confident that I could visit a clinic to get an HIV test**   - Not At All True - A Little True - Mostly True - Completely True |
|  | **I am confident that I could access condoms and other forms of contraception**   - Not At All True - A Little True - Mostly True - Completely True |
|  | **I am confident that I would be able to go to a health clinic for any reason, if I wish to.**   - Not At All True - A Little True - Mostly True - Completely True |
|  | **Entertainment** |
|  | **How often do you watch TV series?**   - Almost every day, - At least once a week, - Less than once a week - Not at all |
|  | **Which channel do you usually watch TV series on?**   - SABC 1 - SABC 2 - SABC 3 - ETV - DSTV channel - Other |
|  | **Do you have access to the internet via a cell phone , computer , tablet etc.?**   - No - Yes |
|  | **Some people use instant messaging services or social media such as Whatsapp or Facebook to chat to friends.**  **Have you used any instant messaging services in last 12 months?**   - No - Yes |
|  | **What instant messaging services or social media do you use?**   - Mxit - Whatsapp - BBM (BlackBerry messenger) - Snapchat - Instagram - Facebook - Twitter - Other___________ |
|  | **Have you made any new male friends through any of these social media platforms in last 12 months?**   - No - Yes |
|  | **How many new male friends from online have you physically met and spent time with in last 12 months?**   - Close to my age ____________ - >5 years and older___________ |
|  | **Have you had a relationship with any of the older men (>5 years) you met online?**   - No - Yes |
|  | **Recreation / Socialise** |
|  | **Where do you normal socialise at night  (choose more than one)**   - Community/School field where young people hand out - A shebeen/tavern/ Shisa Nyama/ - A night club/disco - A sport club - Mavuso stokvel parties - The mall to see a movie or restaurant - Church meetings - Stay home - Other ______________________ |
|  | **Substance abuse** |
|  | **How many of your friends drink alcohol at least once a week?**   - All - Some - None - Refused |
|  | **How many of your friends use dagga at least once a week? (also known as marijuana, weed, grass)**   - All - Some - None - Refused |
|  | **How often in the past 12 months have you had a drink containing alcohol such as beer, wine, spirits or sorghum beer?^^[[24]](#footnote-24)^^**   - Never [next section] - Monthly or less - 2-4 times a month - 2-3 times a week - 4 + times a week |
|  | **How many drinks did you have on a typical day when you were drinking in the past 12 months?**   - None I do not drink - 1 OR 2 - 3 OR 4 - 5 OR 6 - 7, 8 OR 9 - 10 OR MORE |
|  | **How often did you have six or more drinks on one occasion in the past 12 months?**   - Never - Less than monthly - Monthly - Weekly - Daily or almost daily |
|  | **In the past 12 months, how often have you used any of the following substances?^^[[25]](#footnote-25)^^**  **Cannabis (dagga, glue, tik, XTC, marijuana, pot, grass, hash, etc.)**   - Never - Less than monthly - Monthly - Weekly - Daily or almost daily |
|  | **Cocaine (coke, rocks, crack, etc.) or Amphetamine-type stimulants (speed, XTC, tik, etc.)**   - Never - Less than monthly - Monthly - Weekly - Daily or almost daily |
|  | **Inhalants (nitrates, glue, petrol, paint thinners, etc.)**   - Never - Less than monthly - Monthly - Weekly - Daily or almost daily |
|  | **Sedatives or sleeping pills (Valium, Mandrax, Serepax, Rohypnol, etc.)**   - Never - Less than monthly - Monthly - Weekly - Daily or almost daily |
|  | **Hallucinogens (LSD, acid, mushrooms, PCP, Special K, etc.), Opiates (heroin, morphine, methadone, codeine, etc.) or Whoonga (mixture of heroin, dagga and ARVs), Nyaope**   - Never - Less than monthly - Monthly - Weekly - Daily or almost daily |
|  | **Sexual Activity** |
|  | **First Sex** |
|  | **What is your current relationship or marital status?**   - Single (not in a relationship) - Married (living with husband/wife) - Married (not living with husband/wife) - Living together, not married (living with boyfriend/girlfriend/partner) - Going steady (in a relationship, but not living together) - Divorced / separated - Widower / Widow - Civil Union - Other |
|  | **How many of your friends are sexually active?**   - All - Some - None - Don’t know |
|  | **How many of your friends use condoms when they have sex?**   - All - Some - None - Don’t know |
|  | **How many of your friends pressure you to have sex?**   - All - Some - None - Refused |
|  | **Have you ever had a boyfriend or partner, even if only for a short time?**   - Yes - No - Don’t know |
|  | **Have you ever had sex with a boy or man, even if only once?**   - Yes - No - Don’t know |
|  | **Have you ever had sex with a girl or women, even if only once?**   - Yes - No - Don’t know |
|  | **If ever had sex: At what age did you first have sex?**  ___Age in Years |
|  | **If never had sex: Could you please tell me why you have not had sex yet?**   - Not ready - I am too young - Not interested - Avoiding pregnancy - Avoiding STDs, including HIV - Religious grounds - Cultural grounds - Don't have a partner - No response - Other   **(skip to next section)** |
|  | **Who was your first sexual partner?**   - Main partner/boyfriend - khwapeni (casual partner) - once-off sexual partner - Other _________ |
|  | **What was their age _____** |
|  | **The first time you had sex , were you forced to have sex?**   - Yes - No - Refused |
|  | **The first time you had sex did you use a condom?**   - Yes - No - Refused |
|  | **Last sexual partner** |
|  | **Who was your LAST sexual partner?**   - Main partner/boyfriend - khwapeni (casual partner) - once-off sexual partner - Other _________ |
|  | **Age ___________** |
|  | **The last time you had sex did you use a condom?**   - Yes - No - Refused |
|  | **The last time you had sex, was the partner you had sex with circumcised?**   - Yes - No - Refused |
|  | **Was your last partner HIV positive?**   - Yes - No - I don’t know - Refused |
|  | **If positive: Was this partner taking ARVs for HIV?**   - Yes - No - I don’t know - Refused |
|  | **Sexual Partners** |
|  | **How many partners have you had sex with in the last 12 months?**   - Main partners/boyfriends [number] - khwapeni (casual partners) [number] - once-off sexual partners [number] |
|  | **In the whole of your life, including the last 12 months, how many partners have you had sex with?**   - Main partners/boyfriends [number] - khwapeni (casual partners) [number] - once-off sexual partners [number] |
|  | **How many sexual partners have you had in total?**   - Number |
|  | **In last 12 months** |
|  | **If sex with any partner in past 12 months:**  **How often in the last 12 months did you consume alcohol or drugs before having sex?**   - Always - Often - Sometimes - Never |
|  | **How often have you used condoms in the last 12 months when you had sex?**   - Always - Often - Sometimes - No use |
|  | **In the past 12 months have any of your sexual partners been five or more years older than you?**   - Yes - No |
|  | **Think about any man you have had sex with in the past 12 months, whether main partner or casual or once-off partner. Did you have sex with them or stay in a relationship with them so that you could receive any of the following:**   - Cash or money to be looked after - Somewhere to stay or sleep for the night - Support or money for your family - Help with food, paying bills, or school fees - Cosmetics, clothes, a cell phone, airtime, transportation or anything else you couldn’t afford by yourself |
|  | **Sexual Self efficacy (skip if never had sex)** |
|  | **Do you feel able to:**  **Refuse sex with a partner if you did not feel like having sex?^^[[26]](#footnote-26)^^**   - Yes - No - Maybe |
|  | **Refuse sex with a partner who offers or buys you gifts**   - Yes - No - Maybe |
|  | **Refuse sex with a partner who did not want to use a condom when you did.**   - Yes - No - Maybe |
|  | **Refuse sex if your partner was drunk and you did not want sex.**   - Yes - No - Maybe |
|  | **Use a condom correctly?**   - Yes - No - Maybe |
|  | **Put a condom on your partner?**   - Yes - No - Maybe |
|  | **Convince a new partner to use condoms if you want to use a condom?**   - Yes - No - Maybe |
|  | **Ask a partner who you haven’t been using condoms with, to start using them?**   - Yes - No - Maybe |
|  | **Remember to ask a partner to use condom even after you have been drinking?**   - Yes - No - Maybe |
|  | **Get a partner to use condoms, even if he was drunk or high?**   - Yes - No - Maybe |
|  | **STIS (skip never had sex)** |
|  | **STIs**  **Has a doctor or nurse ever told you that you have a sexually transmitted infection (STI) in last 12 months (e.g. discharge or bleeding from vagina; genital ulcers, swelling or irritation; difficulty in urinating; painful intercourse; pain in uterus)?**   - Yes - No - Refused |
|  | **If yes: Did you complete your treatment for your STI?**   - Yes - No - Refused |
|  | **If yes, who provided it?**   - no-one - self-medicated expecting to heal - Traditional healer - Health care clinic - Private doctor - Clinic staff - Don't know - Refused |
|  | **Did your symptoms clear up completely after treatment?**   - Yes - No - Refused |
|  | **Financial aspirations in a relationship** |
|  | **If you spent the night with someone and had sex with them, would you expect to receive a gift of money in the morning to spend on clothes/shoes/weaves ect…?**   - Yes - No |
|  | **Would you expect someone you are having a sexual relationship with to give you money for clothes/shoes/weaves ect…?**   - Yes - No |
|  | **If you had a sexual relationship with an older man (+-10 years older) , would you expect him to give you gifts of money to spend on clothes/shoes/weaves ect…**   - Yes - No |
|  | **If you had a sexual relationship with a married man , would you expect him to give you gifts of money to spend on clothes/shoes/weaves etc.…**   - Yes - No |
|  | **I am now going to ask you a few question on the practice that has become known as ‘blessing’ in South Africa. We define blessings as a relationships in which one partner (the ‘blesser’) spoils the other (the ‘blessee’) with gifts in exchange for sexual favours. The value of the gifts may vary and come in the form of food, airtime and car lifts to fashionable clothes, cars and trips.** |
|  | **Have you heard about this practice “Blessing” in your community?**   - Yes - No > skip to next section |
|  | **Who are blessors in your community?**   - Teacher - Preacher/Priest - Taxi Driver - Older man from a club/pub - Male family friends - Other **_______** |
|  | **Are any of your friends involved in such relationships?**   - Yes - No - Don’t know |
|  | **Have you ever had a blessor relationship?**   - Yes - No |
|  | **How many men have you had blessor relationship with?**   - Number ________ |
|  | **Have you ever had sex with a blessor?**   - Yes - No |
|  | **How old where you when you had your first sexual relationship with a blessor?**   - Age _______ |
|  | **How old was your blesser?**   - Around my age - +-5 years older - +- 10 years older |
|  | **How many blessors have you had sex with?**   - Number ________ |
|  | **In your last sexual act with a blessor did he treat you with kindness and respect?**   - Yes - No |
|  | **In your last sexual act with a blessor did you consume alcohol?**   - Yes - No |
|  | **In your last sexual act with a blessor was a condom used?**   - Yes - No |
|  | **In your last sexual act with a blessor did you use any other form of contraception to protect you from becoming pregnant?**   - Yes - No |
|  | **Do you know if your blesser is blessing any other girls besides you?**   - Yes - No |
|  | **Relationship control** |
|  | **Note: if never had boyfriend/partner – neither romantic/intimate nor sexual partner - SKIP to next section (asked of girls who have a boyfriend/partner but have not had sex):^^[[27]](#footnote-27)^^**  **Thinking about your current/most recent partner or a boyfriend.**  **Most of the time, we do what my partner wants to do.**   - Strongly agree - Agree - Disagree - Strongly disagree |
|  | **My partner won’t let me wear certain things.**   - Strongly agree - Agree - Disagree - Strongly disagree |
|  | **When my partner and I are together, I’m pretty quiet.**   - Strongly agree - Agree - Disagree - Strongly disagree |
|  | **My partner has more say than I do about important decisions that affect us.**   - Strongly agree - Agree - Disagree - Strongly disagree |
|  | **My partner tells me who I can spend time with.**   - Strongly agree - Agree - Disagree - Strongly disagree |
|  | **If I asked my partner to use a condom, he would think I’m having sex with other people.**   - Strongly agree - Agree - Disagree - Strongly disagree |
|  | **My partner does what he wants, even if I do not want him to.**   - Strongly agree - Agree - Disagree - Strongly disagree |
|  | **When my partner and I disagree, he gets his way most of the time.**   - Strongly agree - Agree - Disagree - Strongly disagree |
|  | **If my partner wants to have sex, he would expect me to agree.**   - Strongly agree - Agree - Disagree - Strongly disagree |
|  | **My partner always wants to know where I am.**   - Strongly agree - Agree - Disagree - Strongly disagree |
|  | **If I asked my partner to use a condom, he would get angry.**   - Strongly agree - Agree - Disagree - Strongly disagree |
|  | **My partner might be having sex with someone else.**   - Strongly agree - Agree - Disagree - Strongly disagree |
|  | **He lets me know I am not the only partner he could have.**   - Strongly agree - Agree - Disagree - Strongly disagree |
|  | **Violence** |
|  | **Sometimes couples argue , these arguments may get out of control and get violent. The next few questions are about what you have experienced during these arguments.**  **In the last 12 months have you ever experienced any of the following violent acts from your partner?**   - Beat/hit - Slapped - Partner has thrown something at me - Pushed/shoved - Biting - Hit with fist - Hit with an object (eg. broom stick, shambok) - Kicked - Dragged - Chocked - Burnt - Threatened with weapon (eg. gun, knife) |
|  | **Sexual violence (Skip if never had sex)** |
|  | **In the last 12 months has a current or previous boyfriend or partner**   - **physically forced you to have sex when you did not want to?** - **ever used threats or intimidation to get you to have sex when you did not want to?** - **partner force you to do something else sexual that did not want to do** |
|  | **In the last 12 months has any man who is NOT your boyfriend or partner**   - **forced or persuaded you to have sex against your will?** - **tried to force you to have sex against your will and did not succeed** - **ever forced you to have sex against your will when you were too drunk or drugged to refuse** |
|  | **In the last 12 months how many times did two or more men**   - **force you to have sex with them at the same time against your will?** - **force you to have sex with them at the same time against your will when you were too drunk or drugged to refuse?** |
|  | **If any of the above physical or sexual violence questions are answered with ‘yes’: The last time you experienced this violence did you go to any of the following for help? (multiple answers)**   - Police - Hospital/Health service - Social services - Legal advice centre - Court - Shelter - Local leader - Women’s organisation - Pries/religious leader - Thuthuzela Care Centre/ - TCC |
|  | **Exposure in dream activity** |
|  | **Education at school**  **In the last 12 months, how many times did you receive sexuality education at school?**   - Insert Number of times_____[0 – X] |
|  | **In the last 12 months, how many times did you receive sexuality education at school?**   - Insert Number of times_____[0 – X] |
|  | **Condon use**  **In the last 12 months, how many times did you receive training/education on the use of condoms?**   - Insert Number of times_____[0 – X] |
|  | **In the last 12 months, how many times did you obtain free condoms?**   - Insert Number of times_____[0 – X] |
|  | **Is there somewhere you feel comfortable going to access free condoms?**   - No - Yes |
|  | **In the last 12 months, how many times have you been exposed to HIV testing campaigns?**   - Insert Number of times_____[0 – X] |
|  | **HIV testing & linkage to care**  **In the last 12 months, how many times have you received training/education on the benefit of HIV testing and getting to know your status?**   - Insert Number of times_____[0 – X] |
|  | **In the last 12 months how many times have you been exposed to contraceptive use campaigns?**   - Insert Number of times_____[0 – X] |
|  | **Contraceptives**  **In the last 12 months, how many times have you received training/education on the use of contraceptives and the different types?**   - Insert Number of times_____[0 – X] |
|  | **Youth friendly clinics**  **Are the health workers at the nearest health facility friendly to you?**   - Yes - No - Not sure |
|  | **Safe place**  **In your community, is there a place you can go to regularly to meet with your friends where you feel safe?**   - Yes - No |
|  | **Have you participated in support group programmes in which people your age meet?**   - Yes - No |
|  | **Interview** |
|  | **Apart from you and the interviewee, was anyone else present in the interview or listening to any of the questions?**   - Yes - No |

# Cross-Sectional Study Questionnaire (12-17 years)

**Adolescent Questionnaire**

**12 to 17 years of age**

**Study Title: DREAMS impact evaluation -**

**HIV incidence trends among adolescent girls and young women in the eThekwini (KZN), uMgungundlovu (KZN), City of Johannesburg (GP) and Ekurhuleni (GP) districts, South Africa**

**Version 7.0 20 December 2016**

Since you have agreed to participate in the DREAMS Implementation Evaluation Survey, we would like to ask you a few questions that will help us better understand the impact of the impact of DREAMS programme.

Please feel free to ask any questions that you might have about what we would be asking you.

We will focus on questions on

- Generally about yourself, your family and your environment
- Some of your feelings, thoughts and hopes for the future
- Some behaviours including sexual behaviours that might be predisposing you to HIV
- Where you access health care
- About HIV and AIDS

We hope that you would answer all the questions. Please note that your name will not appear anywhere on the questionnaire, your answers will be kept private and no one will know that the answers came from you. We hope that you answer the questions truthfully and to the best of your ability. Once again please feel free to ask any questions you might feel are important to know.

|  | **Section: Pre program mobile database** |
| --- | --- |
|  | Staff ID  Household ID number  GPS Co-ordinates  Location  Map  Date |
|  | **Section: Participant Criteria** |
|  | **Eligibility**  **Not eligible if answered yes to any of the following questions:**   - Younger than 12 or older than 24 years of age. - Non-resident from the study area. - Refusal by participant to participate in the study. (No consent.) - Refusal by participant to provide clinical samples of DBS/peripheral blood and self-collected vulvo-vaginal swab samples. - Cognitive or mental challenges (based on the assessment of the participant’s ability to comprehend the study information provided). - Deaf or mute. - Not speaking English, IsiZulu, Sotho, Afrikaans. |
|  | **2nd/3rd survey:**   - Have not been living in the area in the past year (2nd survey)/past 2 years (3rd survey). |
|  | **Refusal**  **What are the reasons that you do not want to participate?**   - Participant declined to give a reason(s) for refusal - I don’t have time to participate in the survey - I already know I am HIV positive - I don’t wish to be retested for HIV - I find the topics uncomfortable or embarrassing - I don’t want my samples taken - Other (specify) _______________ |
|  | **Consent**   - Parental / guardian consent - Assent |
|  | **Consent for sample storage and future testing**   - Parental / guardian consent - Assent |
|  | **Sample collection**  **Barcode**   - Scan Barcode |
|  | **Sample collection:**   - Blood sample - Self collected Vulvo-vaginal swabs |
|  | **Demographics** |
|  | **How old were you at your last birthday? ^^[[28]](#footnote-28)^^**   - (Age of the respondent) |
|  | **Information**  **What is your home language?**   - Zulu - Xhosa - Sotho - Tswana - English - Afrikaans - Other ______________. |
|  | **Race of the respondent**   - African - White - Coloured - Indian/Asian - Other |
|  | **What is your nationality?**   - South African citizen - Non-citizen (Permanent resident ) - Non-citizen (Refugee) - Other |
|  | **Mobility**  **How long have you lived in this community?**   - > 5 years - 1-5 years - <1 year |
|  | **In the past 12 months have you been away from your usual residence for more than one month?**   - Yes - No |
|  | **In the past week, how many nights have you stayed away from home?**   - ______ Number of days |
|  | **Education** |
|  | **Do you currently attend school?**   - Yes - No |
|  | **What is your highest school qualification achieved?**   - Grade R - Sub a/class 1/grade 1 - Sub b/class 2/grade 2 - Standard 1/grade 3 /ABET 1 - Standard 2/grade 4 / ABET 2 - Standard 3/grade 5/ABET 2 - Standard 4/grade 6/ABET 3 - Standard 5/grade 7/ABET 3 - Standard 6/grade 8/ABET 3 - Standard 7/grade 9/ABET 3 - Standard 8/grade 10/NTC 1 - Standard 9/grade 11/NTC 2 - Standard 10/grade 12/NTC 3 - Further studies incomplete (skip to employment) - Diploma/other post school completed (skip to employment) - Further degree completed (skip to employment) - Don’t know - No schooling (skip to employment) |
|  | **If not: Why not?**   - My family does not have enough money - I don’t like school - I have to look after my younger brothers/sisters - I have to look after a sick family member - I failed - I was expelled - I became pregnant (if female) - Poor health - Completed grade 12 (skip to employment) - Other |
|  | **If yes: Are you repeating this grade?**   - Yes - No |
|  | **If yes: Why?**   - My family did not have enough money - I don’t like school - I had to look after my younger brothers/sisters - I had to look after a sick family member - I failed - I was expelled - I was pregnant / had a baby / had a child (if female) - I had been sick - Other |
|  | **If attending school: In the last month, have you missed any days of school? (excluding holidays)^^[[29]](#footnote-29)^^**   - Yes: How many days? - No |
|  | **If yes: Why have you missed school?^^[[30]](#footnote-30)^^**   - I have been sick - I don’t feel safe going to school - I don’t feel safe at school - I don’t like school food - I have to look after my younger brothers/sisters - I have to look after a sick family member - I don’t have enough money to go to school everyday - Exams were done - I am pregnant/have a baby/have a child - Other |
|  | **If yes, Family members/carers think that performing well in school is very important^^[[31]](#footnote-31)^^**   - Strongly disagree - Disagree - I don’t agree or disagree - Agree - Strongly agree |
|  | **Employment** |
|  | **How would you describe your present employment situation?**   - Housewife, homemaker, not looking for work - Housewife, homemaker, looking for work - Unemployed, looking for work - Unemployed, not looking for work - Work in informal sector, looking for permanent work - Work in informal sector, not looking for permanent work - Sick/disabled and unable to work - Student/pupil/learner - Self-employed - full time (40 hours or more per week) - Self-employed - part time (less than 40 hours per week) - Employed part time (if none of the above) (less than 40 hours per week) - Employed full time (40 hours or more per week) - Other |
|  | **If employed, considering all the money you earned from jobs or selling things, how much did you earn in the last 4 weeks (not including grants)?**   - Numeric response |
|  | **Do you save money? For example, putting some money aside for another day or for a special purpose?**   - Yes: How much do you have in savings? R - No - Refused |
|  | **If in school: Do you receive money from a program to stay in school?**   - Yes, total amount R - No - Refused |
|  | **Health** |
|  | **At any time in the last two weeks, have you been too sick to participate in daily activities? ^^[[32]](#footnote-32)^^**   - Yes - No |
|  | **Do you have difficulty seeing, even if wearing glasses?^^[[33]](#footnote-33)^^**   - No difficulty - Yes – some difficulty - Yes – a lot of difficulty - Cannot do at all |
|  | **Do you have difficulty hearing, even if using a hearing aid?**   - No difficulty - Yes – some difficulty - Yes – a lot of difficulty - Cannot do at all |
|  | **Do you have difficulty walking or climbing steps?**   - No difficulty - Yes – some difficulty - Yes – a lot of difficulty - Cannot do at all |
|  | **Do you have difficulty remembering or concentrating?**   - No difficulty - Yes – some difficulty - Yes – a lot of difficulty - Cannot do at all |
|  | **Do you have difficulty remembering or concentrating?**   - No difficulty - Yes – some difficulty - Yes – a lot of difficulty - Cannot do at all |
|  | **Do you have difficulty speaking?**   - No difficulty - Yes – some difficulty - Yes – a lot of difficulty - Cannot do at all |
|  | **Psychological health** |
|  | **We would like you to describe way that you may have felt or behaved during the last week:^^[[34]](#footnote-34)^^**  **I felt depressed**   - Rarely (Less than 1 day) - Some of the time (1-2 days) - Occasionally (3-4 days) - All of the time (5-7 days) |
|  | **My sleep was restless**   - Rarely (Less than 1 day) - Some of the time (1-2 days) - Occasionally (3-4 days) - All of the time (5-7 days) |
|  | **I had crying spells**   - Rarely (Less than 1 day) - Some of the time (1-2 days) - Occasionally (3-4 days) - All of the time (5-7 days) |
|  | **I felt lonely**   - Rarely (Less than 1 day) - Some of the time (1-2 days) - Occasionally (3-4 days) - All of the time (5-7 days) |
|  | **I could not get going**   - Rarely (Less than 1 day) - Some of the time (1-2 days) - Occasionally (3-4 days) - All of the time (5-7 days) |
|  | **Resilience** |
|  | **I am able to solve problems without harming myself or others**.^^[[35]](#footnote-35)^^   - Not at all - A little - Somewhat - Quite a bit - A lot |
|  | **I know where to go in the community to get help.**   - Not at all - A little - Somewhat - Quite a bit - A lot |
|  | **Getting an education is important to me.**   - Not at all - A little - Somewhat - Quite a bit - A lot |
|  | **I try to finish what I start.**   - Not at all - A little - Somewhat - Quite a bit - A lot |
|  | **I have people I look up to.**   - Not at all - A little - Somewhat - Quite a bit - A lot |
|  | **My parents/caregivers know a lot about me.**   - Not at all - A little - Somewhat - Quite a bit - A lot |
|  | **My family stands by me during difficult times.**   - Not at all - A little - Somewhat - Quite a bit - A lot |
|  | **My friends stand by me during difficult times.**   - Not at all - A little - Somewhat - Quite a bit - A lot |
|  | **I have opportunities to develop skills that will be useful later in life.**   - Not at all - A little - Somewhat - Quite a bit - A lot |
|  | **I am treated fairly in my community.**   - Not at all - A little - Somewhat - Quite a bit - A lot |
|  | **I feel I belong in my / community**   - Not at all - A little - Somewhat - Quite a bit - A lot |
|  | **I enjoy my cultural and family traditions.**   - Not at all - A little - Somewhat - Quite a bit - A lot |
|  | **Caregiver Relationships** |
|  | **We are now going to ask you some questions about your caregiver. By caregiver, we mean a parent, legal guardian or other adult who is primarily responsible for your well-being; specifically, the person that prepares meals seeks medical attention when you are ill and otherwise cares for you while you were growing up.**  **Who would you say was your primary caregiver currently?**   - Mother - Father - Grandmother - Grandfather - Aunt - Uncle - Sister - Brother - Other - Relative - Non-relative |
|  | ***The next set of questions asks you about your relationship with your caregiver. Remember that your answers are confidential and we will not share them with your caregiver. For each statement, please tell us how true it is for you. The responses are "NOT AT ALL TRUE" "A LITTLE TRUE" "MOSTLY TRUE" OR "COMPLETELY TRUE"^^[[36]](#footnote-36)^^***  **Your [Caregiver] respected your feelings.**   - Not at all true - A little true - Mostly true - Completely true |
|  | **You feel your [Caregiver] did a good job as a caregiver.**   - Not at all true - A little true - Mostly true - Completely true |
|  | **Your [Caregiver] accepted you as you are.**   - Not at all true - A little true - Mostly true - Completely true |
|  | **You liked to get your [Caregiver]’s point of view on things you were concerned about.**   - Not at all true - A little true - Mostly true - Completely true |
|  | **Your [Caregiver] could tell when you were upset about something.**   - Not at all true - A little true - Mostly true - Completely true |
|  | **When you discussed things, your [Caregiver] cared about your point of view.**   - Not at all true - A little true - Mostly true - Completely true |
|  | **Your [Caregiver] trusted your judgment.**   - Not at all true - A little true - Mostly true - Completely true |
|  | **Your [Caregiver] helped you to understand yourself better.**   - Not at all true - A little true - Mostly true - Completely true |
|  | **You told your [Caregiver] about your problems and troubles**   - Not at all true - A little true - Mostly true - Completely true |
|  | **Your [Caregiver] helped you to talk about your difficulties**   - Not at all true - A little true - Mostly true - Completely true |
|  | **Your [Caregiver] understood you.**   - Not at all true - A little true - Mostly true - Completely true |
|  | **When you were angry about something, your [Caregiver] tried to be understanding.**   - Not at all true - A little true - Mostly true - Completely true |
|  | **You trusted your [Caregiver].**   - Not at all true - A little true - Mostly true - Completely true |
|  | **You could count on your [Caregiver] when you needed to get something off your chest.**   - Not at all true - A little true - Mostly true - Completely true |
|  | **If your [Caregiver] knew something was bothering you, they asked you about it.**   - Not at all true - A little true - Mostly true - Completely true |
|  | **Have you spoken to your caregiver in the last 12 months?**   - No - Yes |
|  | **In the last 12 months, how often did your caregiver talk to you about:**  **Unhealthy friendships or relationships?**   - Not at all - Only a little; - Quite a lot - Great deal |
|  | **Talked about HIV/AIDS?**   - Not at all - Only a little; - Quite a lot - Great deal |
|  | **Talked about sex?**   - Not at all - Only a little; - Quite a lot - Great deal |
|  | **Talked about condoms?**   - Not at all - Only a little; - Quite a lot - Great deal |
|  | **Talked about sexually transmitted diseases, or STDs?**   - Not at all - Only a little; - Quite a lot - Great deal |
|  | **Family planning** |
|  | **Are you currently on any contraceptive method?^^[[37]](#footnote-37)^^**   - Yes - No - Refused |
|  | **If no, why not?**   - Trying to have a baby - Cannot access contraceptive methods - Have not thought about getting on it. - Not having sex/abstaining - Currently pregnant - Scared - I am too young - Worried about contraceptive side effects/Experienced contraceptive side effects in the past - Other |
|  | **If yes, Which form(s) of contraceptives are you using?**   - Condoms - Depo-provera (3 month injection) - Nur-isterate (2 month injection) - Oral Pills taken daily - Implant - Intrauterine contraceptive device (IUCD) - Other, specify________ |
|  | **Have you ever been pregnant?**   - Yes - No - Refused |
|  | **If yes, How many times have you been pregnant?**   - [number] |
|  | **If yes, What was your age at your first pregnancy?**   - [number] |
|  | **If yes, were you still at school when you were pregnant?**   - Yes - No - Refused   **If yes, Did you/will you return to school afterwards?**   - Yes - No - Refused |
|  | **If yes, the last time you became pregnant did you:**   - Want to become pregnant then - Want to wait until later to become pregnant - Not want to have any children |
|  | **If yes, what was the outcome of your pregnancy? (each pregnancy)**   - Had termination of pregnancy (TOP) by unqualified person (backdoor abortion). - Had termination of pregnancy (TOP) by clinic staff. - Lost naturally (spontaneously abortion). - Still pregnant. - Live child/ children. |
|  | **If yes, in case of live child/children:**  **Do you receive a child support grant? (per child)**   - Yes - No - Refused |
|  | **Knowledge & perceptions of HIV** |
|  | **Can people reduce their chances of getting HIV (the virus that causes AIDS) by having just one sex partner who is not infected and who has no other partners?’^^[[38]](#footnote-38)^^**   - Yes - No - Don’t know |
|  | **Can people reduce their chances of getting HIV/AIDS by using a condom every time they have sex?**   - Yes - No - Don’t know |
|  | **Can a person get HIV/AIDS from mosquito bites?**   - Yes - No - Don’t know |
|  | **Can people get HIV by sharing food with a person who has HIV/AIDS?**   - Yes - No - Don’t know |
|  | **Is it possible for a healthy-looking person to have HIV/AIDS?**   - Yes - No - Don’t know |
|  | **Can having sex with a virgin cure AIDS?**   - Yes - No - Don’t know |
|  | **Can people get HIV or AIDS because of witchcraft or other supernatural means?**   - Yes - No - Don’t know |
|  | **Have you ever heard of a treatment for HIV – the virus that causes AIDS – called Antiretroviral Therapy (ART)**   - Yes - No - Don’t know |
|  | **If yes: If HIV positive people take Antiretroviral Therapy (ART) can this reduce their risk of transmitting HIV to another person?**   - Yes - No - Don’t know   **Have you ever heard of Pre-exposure Prophylaxis (PrEP)?**   - Yes - No - Don’t know |
|  | **Is a woman's risk of getting HIV reduced if her partner is circumcised?**   - Yes - No - Don’t know |
|  | **Is a woman completely protected from HIV infection if her partner is circumcised?**   - Yes - No - Don’t know |
|  | **HIV status** |
|  | **Do you know of a place nearby where you can get an HIV test?**   - Yes - No |
|  | **Have you ever had an HIV test?**   - Yes - No |
|  | **If yes, How long ago did you have your most recent HIV test?**   - 0 to 6 months - 7 to 12 months - Between 1-2 years ago - Between 2-3 years ago - Three or more years ago |
|  | **If tested in the past 12 months:**  **Where did you get tested?**   - School/university/FET campaign - Community testing campaign - Clinic/hospital - Other |
|  | **If tested in the past 12 months:**  **Did you test together with a partner in the past 12 months?**   - Yes - No - Don’t have a partner |
|  | **Have you been told/informed of the result of your most recent HIV test?**   - Yes - No |
|  | **If never had a test: What are your reasons for not having ever had an HIV test?(multiple answers)**   - I do not know where to get tested - I do not think that I have HIV - I am not at risk for HIV - I trust my partner - I was afraid to find out that I might be HIV positive - I am not ready to have an HIV test - I was concerned about CONFIDENTIALITY - I was concerned about STIGMA, DISCRIMINATION or REJECTION - I was concerned about LOSING MY JOB - I am concerned about the STANDARD OF SERVICE - I haven’t got around to it - Other |
|  | **If ever had a test: What was the result of your latest HIV test (if indicated received)?**   - HIV negative. - HIV positive. - Indeterminate. - Refused. |
|  | **HIV positive only (Skip if HIV negative, indeterminate or refused)** |
|  | **Not including medical personnel or those at the clinic, have you talked to anyone about your HIV status?**   - Nobody - Yes, One person - Yes, More than one person |
|  | **Are you currently being provided with any of the following support?**   - Nutritional support - Emotional support - Treatment buddy - Home based care - Financial support - Treatment of opportunistic infections |
|  | **Have you ever been pregnant while you were HIV positive? (Skip to 130 if never been pregnant )**   - Yes - No |
|  | **If yes, Did your baby become infected with HIV?**   - Yes - No |
|  | **Did you take ART while pregnant to reduce the chances of your child acquiring HIV?**   - Yes - No |
|  | **Risk perceptions** |
|  | **I feel that the chances are high that I can get HIV [skip if already HIV positive]**   - Strongly Agree - Agree - Disagree - Strongly Disagree |
|  | **I believe that older men are more likely to have HIV than younger men.**   - Strongly Agree - Agree - Disagree - Strongly Disagree |
|  | **I feel confident that I could visit a clinic to get an HIV test**   - Not At All True - A Little True - Mostly True - Completely True |
|  | **I am confident that I could access condoms and other forms of contraception**   - Not At All True - A Little True - Mostly True - Completely True |
|  | **I am confident that I would be able to go to a health clinic for any reason, if I wish to.**   - Not At All True - A Little True - Mostly True - Completely True |
|  | **Entertainment** |
|  | **How often do you watch TV series?**   - Almost every day, - At least once a week, - Less than once a week - Not at all |
|  | **Which channel do you usually watch TV series on?**   - SABC 1 - SABC 2 - SABC 3 - ETV - DSTV channel - Other |
|  | **Do you have access to the internet via a cell phone , computer , tablet etc.?**   - No - Yes |
|  | **Some people use instant messaging services or social media such as Whatsapp or Facebook to chat to friends.**  **Have you used any instant messaging services in last 12 months?**   - No - Yes |
|  | **What instant messaging services or social media do you use?**   - Mxit - Whatsapp - BBM (BlackBerry messenger) - Snapchat - Instagram - Facebook - Twitter - Other___________ |
|  | **Have you made any new male friends through any of these social media platforms in last 12 months?**   - No - Yes |
|  | **How many new male friends from online have you physically met and spent time with in last 12 months?**   - Close to my age ____________ - >5 years and older___________ |
|  | **Have you had a relationship with any of the older men (>5 years) you met online?**   - No - Yes |
|  | **Recreation / Socialise** |
|  | **Where do you normal socialise at night  (choose more than one)**   - Community/School field where young people hand out - A shebeen/tavern/ Shisa Nyama/ - A night club/disco - A sport club - Mavuso stokvel parties - The mall to see a movie or restaurant - Church meetings - Stay home - Other ______________________ |
|  | **Substance abuse** |
|  | **How many of your friends drink alcohol at least once a week?**   - All - Some - None - Refused |
|  | **How many of your friends use dagga at least once a week? (also known as marijuana, weed, grass)**   - All - Some - None - Refused |
|  | **How often in the past 12 months have you had a drink containing alcohol such as beer, wine, spirits or sorghum beer?^^[[39]](#footnote-39)^^**   - Never [next section] - Monthly or less - 2-4 times a month - 2-3 times a week - 4 + times a week |
|  | **How many drinks did you have on a typical day when you were drinking in the past 12 months?**   - None I do not drink - 1 OR 2 - 3 OR 4 - 5 OR 6 - 7, 8 OR 9 - 10 OR MORE |
|  | **How often did you have six or more drinks on one occasion in the past 12 months?**   - Never - Less than monthly - Monthly - Weekly - Daily or almost daily |
|  | **In the past 12 months, how often have you used any of the following substances?^^[[40]](#footnote-40)^^**  **Cannabis (dagga, glue, tik, XTC, marijuana, pot, grass, hash, etc.)**   - Never - Less than monthly - Monthly - Weekly - Daily or almost daily |
|  | **Cocaine (coke, rocks, crack, etc.) or Amphetamine-type stimulants (speed, XTC, tik, etc.)**   - Never - Less than monthly - Monthly - Weekly - Daily or almost daily |
|  | **Inhalants (nitrates, glue, petrol, paint thinners, etc.)**   - Never - Less than monthly - Monthly - Weekly - Daily or almost daily |
|  | **Sedatives or sleeping pills (Valium, Mandrax, Serepax, Rohypnol, etc.)**   - Never - Less than monthly - Monthly - Weekly - Daily or almost daily |
|  | **Hallucinogens (LSD, acid, mushrooms, PCP, Special K, etc.), Opiates (heroin, morphine, methadone, codeine, etc.) or Whoonga (mixture of heroin, dagga and ARVs), Nyaope**   - Never - Less than monthly - Monthly - Weekly - Daily or almost daily |
|  | **Sexual Activity** |
|  | **First Sex** |
|  | **Many young people have boyfriends. Are you currently dating someone?**   - Yes - No |
|  | **What is your current relationship or marital status?**   - Single (not in a relationship) - Married (living with husband/wife) - Married (not living with husband/wife) - Living together, not married (living with boyfriend/girlfriend/partner) - Going steady (in a relationship, but not living together) - Divorced / separated - Widower / Widow - Civil Union - Other |
|  | **Are your friends currently dating someone?** |
|  | **How many of your friends are sexually active?**   - All - Some - None - Don’t know |
|  | **If friend sexually active,**  **How many of your friends use condoms when they have sex?**   - All - Some - None - Don’t know |
|  | **How many of your friends pressure you to have sex?**   - All - Some - None - Refused |
|  | **Have you ever had a boyfriend or partner, even if only for a short time?**   - Yes - No - Don’t know |
|  | **Have you ever been on a date with a boy or man, even if only once?**   - Yes - No - Don’t know |
|  | **Have you ever kissed a boy or man, even if only once?**   - Yes - No - Don’t know |
|  | **Have you ever had sex with a boy or man, even if only once?**   - Yes - No - Don’t know |
|  | **Have you ever had sex with a girl or women, even if only once?**   - Yes - No - Don’t know |
|  | **If ever had sex: At what age did you first have sex?**  ___Age in Years |
|  | **If never had sex: Could you please tell me why you have not had sex yet?**   - Not ready - I am too young - Not interested - Avoiding pregnancy - Avoiding STDs, including HIV - Religious grounds - Cultural grounds - Don't have a partner - No response - Other   **(skip to next section)** |
|  | **Who was your first sexual partner?**   - Main partner/boyfriend - khwapeni (casual partner) - once-off sexual partner - Other _________ |
|  | **What was their age _____** |
|  | **The first time you had sex , were you forced to have sex?**   - Yes - No - Refused |
|  | **The first time you had sex did you use a condom?**   - Yes - No - Refused |
|  | **Last sexual partner** |
|  | **Who was your LAST sexual partner?**   - Main partner/boyfriend - khwapeni (casual partner) - once-off sexual partner - Other _________ |
|  | **Age ___________** |
|  | **The last time you had sex did you use a condom?**   - Yes - No - Refused |
|  | **The last time you had sex, was the partner you had sex with circumcised?**   - Yes - No - Refused |
|  | **Was your last partner HIV positive?**   - Yes - No - I don’t know - Refused |
|  | **If positive: Was this partner taking ARVs for HIV?**   - Yes - No - I don’t know - Refused |
|  | **Sexual Partners** |
|  | **How many partners have you had sex with in the last 12 months?**   - Main partners/boyfriends [number] - khwapeni (casual partners) [number] - once-off sexual partners [number] |
|  | **In the whole of your life, including the last 12 months, how many partners have you had sex with?**   - Main partners/boyfriends [number] - khwapeni (casual partners) [number] - once-off sexual partners [number] |
|  | **How many sexual partners have you had in total?**   - Number |
|  | **In last 12 months** |
|  | **If sex with any partner in past 12 months:**  **How often in the last 12 months did you consume alcohol or drugs before having sex?**   - Always - Often - Sometimes - Never |
|  | **How often have you used condoms in the last 12 months when you had sex?**   - Always - Often - Sometimes - No use |
|  | **In the past 12 months have any of your sexual partners been five or more years older than you?**   - Yes - No |
|  | **Think about any man you have had sex with in the past 12 months, whether main partner or casual or once-off partner. Did you have sex with them or stay in a relationship with them so that you could receive any of the following:**   - Cash or money to be looked after - Somewhere to stay or sleep for the night - Support or money for your family - Help with food, paying bills, or school fees - Cosmetics, clothes, a cell phone, airtime, transportation or anything else you couldn’t afford by yourself |
|  | **Sexual Self efficacy (skip if never had sex)** |
|  | **Do you feel able to:**  **Refuse sex with a partner if you did not feel like having sex?^^[[41]](#footnote-41)^^**   - Yes - No - Maybe |
|  | **Refuse sex with a partner who offers or buys you gifts**   - Yes - No - Maybe |
|  | **Refuse sex with a partner who did not want to use a condom when you did.**   - Yes - No - Maybe |
|  | **Refuse sex if your partner was drunk and you did not want sex.**   - Yes - No - Maybe |
|  | **Use a condom correctly?**   - Yes - No - Maybe |
|  | **Put a condom on your partner?**   - Yes - No - Maybe |
|  | **Convince a new partner to use condoms if you want to use a condom?**   - Yes - No - Maybe |
|  | **Ask a partner who you haven’t been using condoms with, to start using them?**   - Yes - No - Maybe |
|  | **Remember to ask a partner to use condom even after you have been drinking?**   - Yes - No - Maybe |
|  | **Get a partner to use condoms, even if he was drunk or high?**   - Yes - No - Maybe |
|  | **STIS (skip never had sex)** |
|  | **STIs**  **Has a doctor or nurse ever told you that you have a sexually transmitted infection (STI) in last 12 months (e.g. discharge or bleeding from vagina; genital ulcers, swelling or irritation; difficulty in urinating; painful intercourse; pain in uterus)?**   - Yes - No - Refused |
|  | **If yes: Did you complete your treatment for your STI?**   - Yes - No - Refused |
|  | **If yes, who provided it?**   - no-one - self-medicated expecting to heal - Traditional healer - Health care clinic - Private doctor - Clinic staff - Don't know - Refused |
|  | **Did your symptoms clear up completely after treatment?**   - Yes - No - Refused |
|  | **Financial aspirations in a relationship** |
|  | **If you spent the night with someone and had sex with them, would you expect to receive a gift of money in the morning to spend on clothes/shoes/weaves ect…?**   - Yes - No |
|  | **Would you expect someone you are having a sexual relationship with to give you money for clothes/shoes/weaves ect…?**   - Yes - No |
|  | **If you had a sexual relationship with an older man (+-10 years older) , would you expect him to give you gifts of money to spend on clothes/shoes/weaves ect…**   - Yes - No |
|  | **If you had a sexual relationship with a married man , would you expect him to give you gifts of money to spend on clothes/shoes/weaves ect…**   - Yes - No |
|  | **I am now going to ask you a few question on the practice that has become known as ‘blessing’ in South Africa. We define blessings as a relationships in which one partner (the ‘blesser’) spoils the other (the ‘blessee’) with gifts in exchange for sexual favours. The value of the gifts may vary and come in the form of food, airtime and car lifts to fashionable clothes, cars and trips.** |
|  | **Have you heard about this practice “Blessing” in your community?**   - Yes - No > skip to next section |
|  | **Who are blessors in your community?**   - Teacher - Preacher/Priest - Taxi Driver - Older man from a club/pub - Male family friends - Other **_______** |
|  | **Are any of your friends involved in such relationships?**   - Yes - No - Don’t know |
|  | **Have you ever had a blessor relationship?**   - Yes - No |
|  | **How many men have you had blessor relationship with?**   - Number ________ |
|  | **Have you ever had sex with a blessor?**   - Yes - No |
|  | **How old where you when you had your first sexual relationship with a blessor?**   - Age _______ |
|  | **How old was your blesser?**   - Around my age - +-5 years older - +- 10 years older |
|  | **How many blessors have you had sex with?**   - Number ________ |
|  | **In your last sexual act with a blessor did he treat you with kindness and respect?**   - Yes - No |
|  | **In your last sexual act with a blessor did you consume alcohol?**   - Yes - No |
|  | **In your last sexual act with a blessor was a condom used?**   - Yes - No |
|  | **In your last sexual act with a blessor did you use any other form of contraception to protect you from becoming pregnant?**   - Yes - No |
|  | **Do you know if your blesser is blessing any other girls besides you?**   - Yes - No |
|  | **Relationship control** |
|  | **Note: if never had boyfriend/partner – neither romantic/intimate nor sexual partner - SKIP to next section (asked of girls who have a boyfriend/partner but have not had sex):^^[[42]](#footnote-42)^^**  **Thinking about your current/most recent partner or a boyfriend.**  **Most of the time, we do what my partner wants to do.**   - Strongly agree - Agree - Disagree - Strongly disagree |
|  | **My partner won’t let me wear certain things.**   - Strongly agree - Agree - Disagree - Strongly disagree |
|  | **When my partner and I are together, I’m pretty quiet.**   - Strongly agree - Agree - Disagree - Strongly disagree |
|  | **My partner has more say than I do about important decisions that affect us.**   - Strongly agree - Agree - Disagree - Strongly disagree |
|  | **My partner tells me who I can spend time with.**   - Strongly agree - Agree - Disagree - Strongly disagree |
|  | **If I asked my partner to use a condom, he would think I’m having sex with other people.**   - Strongly agree - Agree - Disagree - Strongly disagree |
|  | **My partner does what he wants, even if I do not want him to.**   - Strongly agree - Agree - Disagree - Strongly disagree |
|  | **When my partner and I disagree, he gets his way most of the time.**   - Strongly agree - Agree - Disagree - Strongly disagree |
|  | **If my partner wants to have sex, he would expect me to agree.**   - Strongly agree - Agree - Disagree - Strongly disagree |
|  | **My partner always wants to know where I am.**   - Strongly agree - Agree - Disagree - Strongly disagree |
|  | **If I asked my partner to use a condom, he would get angry.**   - Strongly agree - Agree - Disagree - Strongly disagree |
|  | **My partner might be having sex with someone else.**   - Strongly agree - Agree - Disagree - Strongly disagree |
|  | **He lets me know I am not the only partner he could have.**   - Strongly agree - Agree - Disagree - Strongly disagree |
|  | **Violence** |
|  | **Sometimes couples argue , these arguments may get out of control and get violent. The next few questions are about what you have experienced during these arguments.**  **In the last 12 months have you ever experienced any of the following violent acts from your partner?**   - Beat/hit - Slapped - Partner has thrown something at me - Pushed/shoved - Biting - Hit with fist - Hit with an object (eg. broom stick, shambok) - Kicked - Dragged - Chocked - Burnt - Threatened with weapon (eg. gun, knife) |
|  | **Sexual violence (Skip if never had sex)** |
|  | **In the last 12 months has a current or previous boyfriend or partner**   - **physically forced you to have sex when you did not want to?** - **ever used threats or intimidation to get you to have sex when you did not want to?** - **partner force you to do something else sexual that did not want to do** |
|  | **In the last 12 months has any man who is NOT your boyfriend or partner**   - **forced or persuaded you to have sex against your will?** - **tried to force you to have sex against your will and did not succeed** - **ever forced you to have sex against your will when you were too drunk or drugged to refuse** |
|  | **In the last 12 months how many times did two or more men**   - **force you to have sex with them at the same time against your will?** - **force you to have sex with them at the same time against your will when you were too drunk or drugged to refuse?** |
|  | **If any of the above physical or sexual violence questions are answered with ‘yes’: The last time you experienced this violence did you go to any of the following for help? (multiple answers)**   - Police - Hospital/Health service - Social services - Legal advice centre - Court - Shelter - Local leader - Women’s organisation - Pries/religious leader - Thuthuzela Care Centre/ - TCC |
|  | **Exposure in dream activity** |
|  | **Education at school**  **In the last 12 months, how many times did you receive sexuality education at school?**   - Insert Number of times_____[0 – X] |
|  | **In the last 12 months, how many times did you receive sexuality education at school?**   - Insert Number of times_____[0 – X] |
|  | **Condon use**  **In the last 12 months, how many times did you receive training/education on the use of condoms?**   - Insert Number of times_____[0 – X] |
|  | **In the last 12 months, how many times did you obtain free condoms?**   - Insert Number of times_____[0 – X] |
|  | **Is there somewhere you feel comfortable going to access free condoms?**   - No - Yes |
|  | **In the last 12 months, how many times have you been exposed to HIV testing campaigns?**   - Insert Number of times_____[0 – X] |
|  | **HIV testing & linkage to care**  **In the last 12 months, how many times have you received training/education on the benefit of HIV testing and getting to know your status?**   - Insert Number of times_____[0 – X] |
|  | **In the last 12 months how many times have you been exposed to contraceptive use campaigns?**   - Insert Number of times_____[0 – X] |
|  | **Contraceptives**  **In the last 12 months, how many times have you received training/education on the use of contraceptives and the different types?**   - Insert Number of times_____[0 – X] |
|  | **Youth friendly clinics**  **Are the health workers at the nearest health facility friendly to you?**   - Yes - No - Not sure |
|  | **Safe place**  **In your community, is there a place you can go to regularly to meet with your friends where you feel safe?**   - Yes - No |
|  | **Have you participated in support group programmes in which people your age meet?**   - Yes - No |
|  | **Interview** |
|  | **Apart from you and the interviewee, was anyone else present in the interview or listening to any of the questions?**   - Yes - No |

1. HSRC [↑](#footnote-ref-1)
2. HSRC [↑](#footnote-ref-2)
3. HSRC [↑](#footnote-ref-3)
4. HSRC [↑](#footnote-ref-4)
5. HSRC [↑](#footnote-ref-5)
6. United Nations Children’s Fund (UNICEF). Guidance document. Developing and operationalizing a national monitoring and evaluation system for the protection, care and support of orphans and vulnerable children living in a world with HIV and AIDS.2009 [cited 2010]. Available from: www.unicef.org/aids/files/OVC_MandE_Guidance_FINAL.pdf. Accessed 3 Sept 2014. [↑](#footnote-ref-6)
7. Washington group http://www.cdc.gov/nchs/washington_

   group/wg_questions.htm [↑](#footnote-ref-7)
8. Center for Epidemiologic Studies Depression Scale (CES-D) 5 items: Shrout and Yagar, 1989 [↑](#footnote-ref-8)
9. Supportive scale of the behaviour inventory [↑](#footnote-ref-9)
10. Supportive scale of the behaviour inventory [↑](#footnote-ref-10)
11. Pact study [↑](#footnote-ref-11)
12. short form Brief Problem Monitor – Achenbach [↑](#footnote-ref-12)
13. HSRC 2016 [↑](#footnote-ref-13)
14. HRSC [↑](#footnote-ref-14)
15. HRSC [↑](#footnote-ref-15)
16. Strand S, Winston J. Educational aspirations in inner city schools. Educational Studies. 2008;34(4):249-267. [↑](#footnote-ref-16)
17. PEPFAR [↑](#footnote-ref-17)
18. Washington group <http://www.cdc.gov/nchs/washington_>

    group/wg_questions.htm [↑](#footnote-ref-18)
19. Center for Epidemiologic Studies Depression Scale (CES-D) 5 items: Shrout and Yagar, 1989 [↑](#footnote-ref-19)
20. CYRM-12 [↑](#footnote-ref-20)
21. Amsden & Greenberg, 1987. 15 item Positive Connection subscale of the Inventory of Peer & Peer Attachment [↑](#footnote-ref-21)
22. Stepping Stones trial, Jewkes et al 2006 [↑](#footnote-ref-22)
23. HIV/AIDS from DHS—except for Q no 6 [↑](#footnote-ref-23)
24. AUDIT C [↑](#footnote-ref-24)
25. HSRC [↑](#footnote-ref-25)
26. adapted from the SATZ Women’s Questionnaire [6] and the Condom Use Self-Efficacy Scale (CUSES) [8] [↑](#footnote-ref-26)
27. Sexual Relationship Power Scale [↑](#footnote-ref-27)
28. HSRC 2016 [↑](#footnote-ref-28)
29. HRSC [↑](#footnote-ref-29)
30. HRSC [↑](#footnote-ref-30)
31. Strand S, Winston J. Educational aspirations in inner city schools. Educational Studies. 2008;34(4):249-267. [↑](#footnote-ref-31)
32. PEPFAR [↑](#footnote-ref-32)
33. Washington group <http://www.cdc.gov/nchs/washington_>

    group/wg_questions.htm [↑](#footnote-ref-33)
34. Center for Epidemiologic Studies Depression Scale (CES-D) 5 items: Shrout and Yagar, 1989 [↑](#footnote-ref-34)
35. CYRM-12 [↑](#footnote-ref-35)
36. Amsden & Greenberg, 1987. 15 item Positive Connection subscale of the Inventory of Peer & Peer Attachment [↑](#footnote-ref-36)
37. Stepping Stones trial, Jewkes et al 2006 [↑](#footnote-ref-37)
38. HIV/AIDS from DHS—except for Q no 6 [↑](#footnote-ref-38)
39. AUDIT C [↑](#footnote-ref-39)
40. HSRC [↑](#footnote-ref-40)
41. adapted from the SATZ Women’s Questionnaire [6] and the Condom Use Self-Efficacy Scale (CUSES) [8] [↑](#footnote-ref-41)
42. Sexual Relationship Power Scale [↑](#footnote-ref-42)
